# Bayesian multiplex network models in R using STRAND: methods for biologists and social scientists

**DOI:** 10.1098/rsos.250555

**Published:** 2025-10-22

**Authors:** Cody T. Ross, Kotrina Kajokaite, Sean Pinkney, Sebastian Sosa

**Affiliations:** ^1^ Department of Human Behavior, Ecology and Culture, Max-Planck-Institut fur Evolutionare Anthropologie, Leipzig, Sachsen, Germany; ^2^ Center of Excellence, Omnicom Group Inc., New York, NY, USA

**Keywords:** multiplex models, social networks, social relations, R software

## Abstract

The social networks of interest to biologists, ecologists and social scientists are often multi-layered, with the same set of individuals interacting with one another in complex, multifaceted ways. Each type of interaction can be represented as one layer in a larger multiplex network. Important research questions often hinge on how ties or flows in one network layer impact ties or flows in another layer. Similar questions focus on the relationship between nodal characteristics across layers: for example, is an individual with a high out-degree in one layer more likely to have a high in-degree in another layer? These questions are effectively addressed by multiplex extensions of generative social network models, like the social relations model (SRM). Here, we present a multiplex implementation of the SRM in the STRAND R package and provide tutorials teaching end-users how to run the model on their own data. We provide worked examples of data analysis, parameter visualization and results interpretation, using datasets from experimental economics and animal behaviour. Our software package allows end-users to deploy powerful multiplex models, using only simple base-R model syntax, permitting wider use of generative network modelling approaches across disciplines.

## 1. Introduction

Complex systems—i.e. groups of interacting entities—are found at microscopic and macroscopic levels—e.g. in protein interactions in cells [1], in populations of microorganisms [2], in human and animal social networks [3–5], in transportation or communication systems [6] and in higher-level social and economic organizations [7]. Such systems are characterized by numerous interconnections (often called ties or edges) between the elements of the system (often called nodes or vertices), which make each element of the system highly interdependent (i.e. if one element is affected by an external event, it is likely that linked elements will be as well). This interdependence complicates the application of standard statistical tools to empirical datasets gleaned from complex systems (see [8,9] for a review).

Because complex systems appear in a wide variety of fields, the commonalities among them have become a focus of cross-disciplinary research (e.g. in complex systems theory and network science [9–11]). As an interdisciplinary domain, the study of complex systems has been heavily influenced by researchers in applied statistics [8,12], who have developed a suite of generative models—e.g. the social relations model (SRM) [13–15], stochastic block models [16,17] and models of collective movement [18]—that can be used to understand the linkages between elements in complex systems [19], as well as study how higher-level phenomena emerge from the simple behaviour of constituent elements [9,18].

Network models have become an essential tool for studying complex systems. The most commonly used statistical methods (e.g. ERGMs, permutation methods and the SRM [4,8–10]) and most empirical work [20], however, focus on understanding the structure of single-layer networks (e.g. [21]). In single-layer networks, an adjacency matrix is used to mathematically represent the existence of connections (e.g. in a binary network), the strength of connections (e.g. in a weighted, undirected network) or directed flows (e.g. in a weighted, directed network) between elements of a system (e.g. proteins, cells, animals, people, cities, etc.). This matrix representation of a network permits the simultaneous evaluation of connection/edge-level features (e.g. reciprocity, transitivity, etc.), node/vertex-level features (e.g. degree heterogeneity, centrality, etc.) and features related to the network as a whole (e.g. sub-group structure, resilience, efficiency of information transmission, etc.) [22]. Network analysis models have played an essential role in developing our understanding of how mechanisms operating at the edge or vertex level create a global system with specific high-level emergent properties.

This being said, most complex systems are not well characterized as single-layer networks [23,24]. For example, individual animals linked in a social network interact with one another in multiple ways; ties between any particular dyad might be positively valenced in one layer (e.g. in food sharing), neutral (e.g. in simple co-residence) or even negatively valenced in another (e.g. during violent aggression events) [25]. Moreover, the actions occurring in one layer may create responses in other layers. Important research questions often hinge on how edge-level events in one network layer impact reciprocal events in another layer [20]. Similarly, higher-level questions focus on the relationship between nodal characteristics across layers: for example, is an individual with a high tendency to act on others in one layer more likely to be acted upon by others in a different layer? Single-layer network theory is not sufficient to describe the structure of such systems, so the paradigm of multiplex network modelling is being actively developed in response to this challenge [20,26–28]. Multiplex networks are simply a set of networks between the same set of nodes, where each network ‘layer’ represents a specific kind of single-layer network connection [29]. By studying the relationships within and between layers of a multiplex network, the questions mentioned above can be effectively addressed, e.g. through multiplex extensions of the SRM [13,30] or conceptually similar models [31].

**Figure 1.**
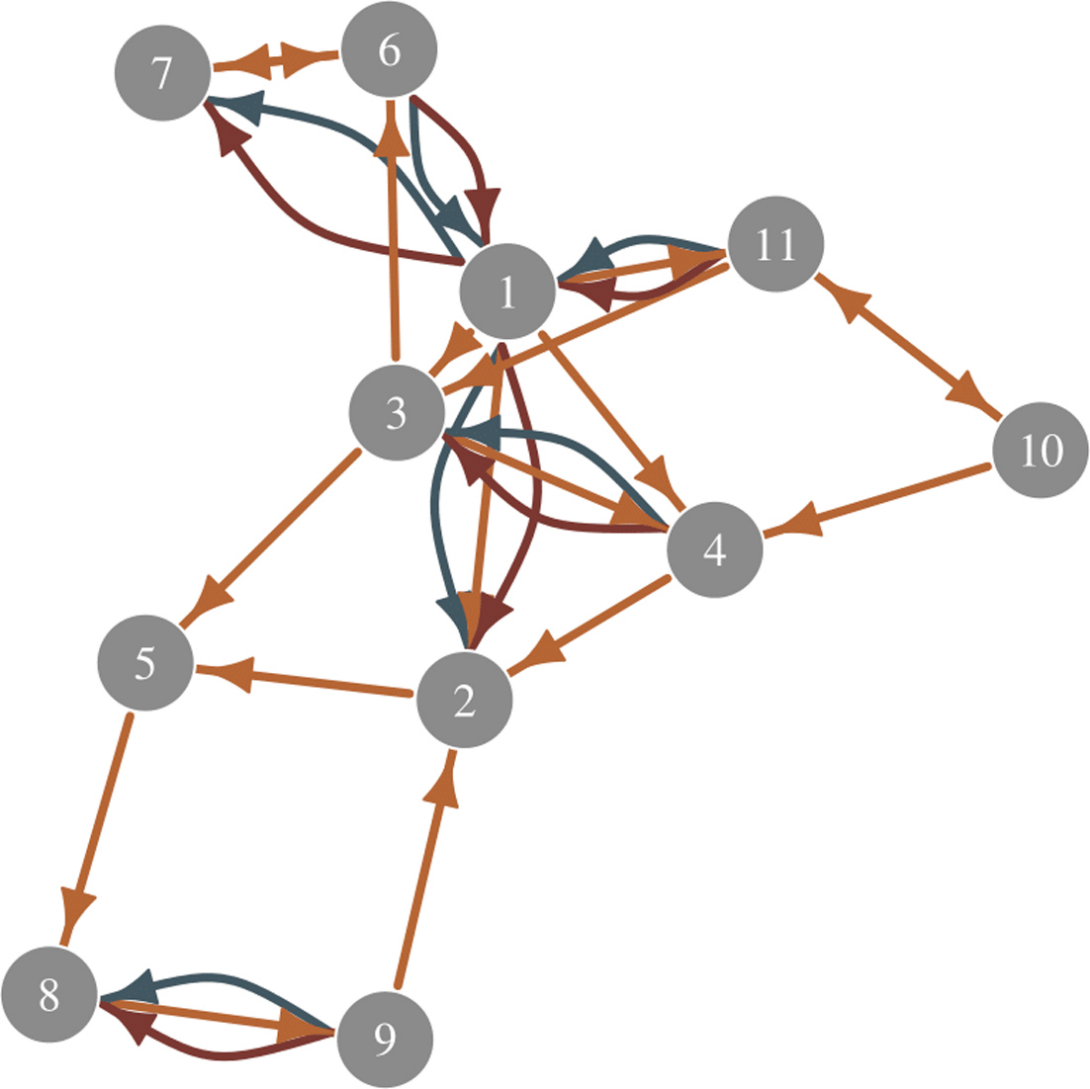
A simple directed multiplex network. Each colour represents a different kind of tie. For example, orange edges might represent grooming events, blue edges might represent threat display events and red edges might represent aggression events. In a single-layer network—e.g. as represented by just the orange ties—analysis typically focuses on the predictors of directed flows, the predictors of variation in nodal in-degree and out-degree and the extent of dyadic reciprocity. In a multiplex network, these factors are still of interest, but estimates are performed within each layer. Additionally, however, within- and between-layer measures of both dyadic and generalized reciprocity are calculated. This allows researchers to study if a tie in one network layer is predictive of a reciprocal tie in another layer, for example, or if an individual’s propensity to have a high out-degree in one layer is predictive of a high in-degree in another layer.

Figure 1 presents a simple visualization of a three-layer multiplex network. To represent M layers of network ties between the same set of N individuals, the standard N×N adjacency matrix can be generalized to a N×N×M array, where each network layer, m, is represented as an N×N sub-array. This mathematical representation makes it possible to estimate within- and between-layer structure at both dyad and node levels using a multi-level correlated random effects structure, as we discuss in detail in §2. Importantly, the M layers of network ties can be defined quite broadly. For example, network layers could represent different types of behaviour (e.g. grooming, food sharing or aggression) between individuals i and j, the locations where behavioural events between i and j occur (e.g. social tolerance ties could be studied in each of M different environment types) or the time periods when ties between i and j occur (e.g. sharing ties could be studied across M different time periods). One can even create combinations of such factors and study the structure of ties within and between such layers—e.g. one can test if friendship ties in time period 1 are predictive of money-lending ties in time period 2.

Although the mathematical properties of multiplex networks have been extensively investigated [24], practical applications of multiplex network analysis models to real-world data remain rare. In the few instances where these models are employed, they typically focus on simple calculations of node metrics across layers [32]. Such descriptive metrics are then incorporated into subsequent, standalone statistical models to assess correlations with other factors of interest. However, this disjointed process prevents the proper propagation of uncertainty through the entire analysis, potentially biasing inference [33]. Although the two-step approach was once viewed as methodologically rigorous, more recent studies have highlighted significant reliability issues [34–37]. In contrast, Bayesian network modelling unifies the estimation of network structure with the assessment of relationships between covariates and network features, all while properly accounting for uncertainty [33]. Accordingly, we introduce a Bayesian data analysis pipeline for processing and modelling multiplex outcomes. Our work complements ongoing efforts aimed at developing robust modelling frameworks for multiplex data (e.g. [31]).

Though theoretical work is progressing rapidly, there is still a dearth of freely available, open-source software tools for implementing Bayesian multiplex network analysis methods using simple, base-R style model specification. To address this gap in the software space, we have expanded the STRAND software package to support multiplex network models. In what remains of the paper, we first outline the mathematical details of the multiplex SRM. Then, we provide a software tutorial, teaching end-users how to both simulate multiplex network datasets and implement multiplex network analysis models on example datasets from experimental economics and animal behaviour. We conclude by presenting the results of unit tests, illustrating that the multiplex STRAND models are well specified, allowing users to recover the parameters used to simulate hypothetical datasets. Our software allows end-users with limited programming experience to easily code complex multiplex network analysis models using only simple base-R syntax.

## 2. Mathematical model definition

In the interest of completeness, we provide a full mathematical description of our multiplex model here, closely following the structure presented in Redhead *et al.* [30]. However, this paper is written primarily as a software tutorial, so end-users not interested in the finer details of the model can skip ahead to the more practical sections.

We assume that a researcher has collected M layers of network data from a sample of N individuals (e.g. biologists may study grooming, threat display and aggression event ties; or social scientists may study loan-giving, advice and socializing ties). Often, there is a question as to whether or not flows in one layer are predictive of flows in another layer, at either a dyadic or generalized level. To address such questions, researchers can use a multiplex generalization (see [30] of the SRM [13,14,38–40]).

### 2.1. The general model

In such a model, our outcome variable of interest, G, is an N×N×M array, with each element, $G_{\left[i,j,m\right]}$, indicating the strength of a tie from focal individual i, to alter j, in network layer m. Depending on the context, $G_{\left[i,j,m\right]}$ might be a Bernoulli random variable with support in {0,1}, a Gaussian random variable with support in ℝ, a Poisson random variable with support in ℕ or a Binomial random variable with support in $\left\{0,\ldots ,E_{\left[i,j,m\right]}\right\}$, where $E_{\left[i,j,m\right]}$ is an exposure variable, giving the upper limit for $G_{\left[i,j,m\right]}$. STRAND supports all four outcome types, but for simplicity of presentation, we describe the Binomial model first.

The final likelihood expression is given by


(2.1) $$G_{\left[i,j,m\right]}∼\text{Binomial}\left(E_{\left[i,j,m\right]},\text{logistic}(\theta_{\left[i,j,m\right]})\right),$$

where $\theta_{\left[i,j,m\right]}$ gives the log-odds of a directed transfer from focal i to alter j in layer m. STRAND uses a logit link by default, since this is more computationally efficient in Stan, and logit models are generally more robust to outliers and misspecification than probit models since they are heavier-tailed [41]. However, some evidence suggests that probits might be slightly better at in-sample prediction for bivariate outcome models [41], like the SRM, and so advocates of probit models can specify a probit link in STRAND if they desire. In our experience (see electronic supplementary material), results are generally equivalent (up to scaling) under logit and probit links, and logit models in Stan are appreciably faster.

We integrate sender, recipient and dyadic random effects, along with any individual or dyadic predictor variables, using the structure of the SRM. The key difference from the basic SRM is that every parameter is indexed by network layer, m, in addition to i and/or j:


(2.2) $$\begin{array}{lcr} & \theta_{\left[i,j,m\right]}=\eta_{\left[m\right]}+\alpha_{\left[i,m\right]}+\beta_{\left[j,m\right]}+\delta_{\left[i,j,m\right]}+\Lambda (i,j,m)+\ldots & \end{array}$$

The intercept term in layer m is $\eta_{\left[m\right]}$. The next term, $\alpha_{\left[i,m\right]}$, is the ‘sender’ or ‘focal’ effect of individual i in layer m; this parameter measures the likeliness of individual i directing ties outwards towards others in layer m. The next term, $\beta_{\left[j,m\right]}$, is the ‘recipient’ or ‘target’ effect of individual j in layer m; this parameter measures the likeliness of individual j receiving ties from others in layer m. The term $\delta_{\left[i,j,m\right]}$ is a dyad-level random effect in layer m; this parameter measures the likeliness of individual i directing a tie to individual j in layer m. The term Λ(i,j,m) is a function giving a dyadic intercept offset as a function of group/block-structuring variables (if they are provided; see details below). Finally, the ellipse in equation (2.2) can be replaced with a linear model for the effects of variables linked to the sender, receiver or dyad. For example, controls for body-size, S, and relatedness, R, could be included by adding: $\kappa_{\left[1,m\right]}S_{\left[i\right]}+\kappa_{\left[2,m\right]}S_{\left[j\right]}+\kappa_{\left[3,m\right]}R_{\left[i,j\right]}$. If such controls are included, the random effects correlations presented later must be interpreted as showing residual correlations, after accounting for the effects of the predictors in each layer.

To model block structure, we can consider a list of V categorical variables—e.g. sex or group—describing individuals i and j. Let $B_{\left[m,v,u,w\right]}$ be a four-dimensional parameter array, where v runs over variables, and u and w run over the category/block levels within variables. Finally, let the function b(i,v) return the block of individual i for variable v. Then, we can define Λ(i,j,m), such that


(2.3) $$\Lambda (i,j,m)=\sum_{v=1}^{V}B_{\left[m,v,b(i,v),b(j,v)\right]},$$

where the probability of a tie from individual i in block b(i,v) to individual j in block b(j,v) for variable v is controlled by the corresponding entry in the array of block parameters, $B_{\left[m,v,b(i,v),b(j,v)\right]}$. From this set of block parameters, we can calculate any of a variety of assortativity coefficients (e.g. [42]), which describe how much individuals tend to associate with others of the same type as themselves.

Next, we model within- and between-layer correlations in network flows at both generalized and dyadic levels, using a multi-level model structure. In the standard SRM, bivariate normal distributions are used to estimate *generalized reciprocity* (i.e. the correlation between sender effects, α, and recipient effects, β) and *dyadic reciprocity* (i.e. the correlation between flows from i to j, $\delta_{\left[i,j\right]}$, and flows from j to i, $\delta_{\left[j,i\right]}$). This approach, however, is insufficient for multiplex data, as there are additional correlations that must be estimated. For example, the correlation of the α parameters in layer l with the α parameters in layer m indicates how likely an individual with a high out-degree in layer l is to also have a high out-degree in layer m. Similarly, the correlation of the α parameters in layer l with the β parameters in layer m indicates how likely an individual with a high out-degree in layer l is to also have a high in-degree in layer m.

The basic SRM sub-model for generalized reciprocity can be extended to account for all of these additional correlations by concatenating the sender and receiver effects for each layer into a single vector and then using a standard multivariate normal model


(2.4) $$\left(\begin{array}{c} \alpha [i,1]\\ \alpha [\dddot{i,M}]\\ \beta [i,1]\\ \beta [\dddot{i,M}] \end{array}\right)∼M.V.\mathrm{Normal}(Z,\Sigma ),$$

where Z is a vector of zeros and Σ is a 2M×2M covariance matrix. Computationally (see [43]), it is much more efficient to implement this model by instead defining


(2.5) $$\left(\begin{array}{c} \alpha_{\left[i,1\right]}\\ \ldots \\ \alpha_{\left[i,M\right]}\\ \beta_{\left[i,1\right]}\\ \ldots \\ \beta_{\left[i,M\right]} \end{array}\right)=\sigma \circ \left(L\left(\begin{array}{c} {\hat{\alpha }}_{\left[i,1\right]}\\ \ldots \\ {\hat{\alpha }}_{\left[i,M\right]}\\ {\hat{\beta }}_{\left[i,1\right]}\\ \ldots \\ {\hat{\beta }}_{\left[i,M\right]} \end{array}\right)\right),$$

where σ is a vector of standard deviation parameters, L is a Cholesky factor of a 2M×2M correlation matrix, ρ, and the symbol ∘ denotes the Hadamard, or element-wise, product. When all of the raw random effects, $\hat{\alpha }$ and $\hat{\beta }$, are given unit-normal priors


(2.6) $$\begin{array}{lcr} & {\hat{\alpha }}_{\left[i,m\right]}∼\text{Normal}(0,1) & \end{array}$$

(2.7) $$\begin{array}{lcr} & {\hat{\beta }}_{\left[i,m\right]}∼\text{Normal}(0,1) & \end{array}$$

then equation (2.5) is equivalent to equation (2.4) [43]. The sub-model is completed by putting weak priors on the standard deviation parameters and the Cholesky factor


(2.8) $$\begin{array}{lcr} & \sigma ∼\text{Exponential}(2.5) & \end{array}$$

(2.9) L∼LKJ Cholesky(2.5).

We can use a similar approach for the dyad-level random effects. In the basic SRM, the dyadic reciprocity coefficient measures the extent to which network flows are bidirectional—i.e. it tests if when an individual i acts on j, that j is more likely to act on i. The multiplex SRM must also test for cross-layer dyadic reciprocity—e.g. it tests if when individual i acts on individual j in layer l, that j is more likely to act on i in layer m.

The dyadic reciprocity sub-model follows a similar form to the generalized reciprocity sub-model; however, there are some additional constraints on the standard deviation and correlation parameters that must be accounted for [30]. First, the dyadic random effects are concatenated across network layers and modelled using a standard multivariate normal model:


(2.10) $$\left(\begin{array}{c} \delta_{\left[i,j,1\right]}\\ \ldots \\ \delta_{\left[i,j,M\right]}\\ \delta_{\left[j,i,1\right]}\\ \ldots \\ \delta_{\left[j,i,M\right]} \end{array}\right)=\varsigma \circ \left(\Gamma \left(\begin{array}{c} {\hat{\delta }}_{\left[i,j,1\right]}\\ \cdots \\ {\hat{\delta }}_{\left[i,j,M\right]}\\ {\hat{\delta }}_{\left[j,i,1\right]}\\ \ldots \cdots \\ {\hat{\delta }}_{\left[j,i,M\right]} \end{array}\right)\right),$$

where ς is a vector of standard deviation parameters and Γ is a Cholesky factor of a 2M×2M correlation matrix, ϱ. The raw random effects, $\hat{\delta }$, have unit-normal priors


(2.11) $${\hat{\delta }}_{\left[i,j,m\right]}∼\text{Normal}(0,1)$$

and weak priors are placed on the standard deviation parameters and the correlation matrix Cholesky factor:


(2.12) $$\begin{array}{lcr} & \varsigma ∼\text{Exponential}(2.5) & \end{array}$$

(2.13) Γ∼LKJ Cholesky(2.5).

Next, we address the special constraints that are needed in the dyadic reciprocity sub-model. First, for a multiplex SRM, the variance of the $\delta_{\left[i,j,m\right]}$ parameters must match the variance of the $\delta_{\left[j,i,m\right]}$ parameters, as the labels i and j are arbitrary. To impose this constraint, we fix ς to use only M free parameters and define the other M parameters by writing


(2.14) $$\varsigma_{\left[m\right]}=\varsigma_{\left[m+M\right]}.$$

Next, the correlation matrix requires a special inner symmetry. Note, for example, that the parameter that measures the correlation between the propensity of individual i to act on to j in layer l and act on j in layer m should be equal to the parameter that measures the correlation between the propensity of individual j to act on to i in layer l and act on i in layer m, as the labels i and j are again arbitrary. Accounting for all such constraints, the dyadic correlation matrix, ϱ, must be of the special block structure


(2.15) $$\varrho =\left(\begin{array}{ll} C & B\\ B & C \end{array}\right),$$

where C is a valid M×M correlation matrix, the elements of B∈(−1,1), and B is equal to its own transpose (B = $B^{T}$). To enforce this condition, we note that ϱ can be generated by multiplying Γ by its own transpose:


(2.16) $$\varrho =\Gamma \Gamma^{T},$$

and then we note that the posterior distribution of ϱ can be forced to take the form given in equation (2.15) by using priors to penalize two particular $\ell^{2}$ norms.

Specifically, for m∈{1,…,M−1} and n∈{m+1,…,M}, we model


(2.17) $$\begin{array}{lcr} & \left\|\;\varrho_{\left[m+M,n+M\right]}-\varrho_{\left[m,n\right]}\;\|∼\text{Normal}(0,\epsilon \right) & \end{array}$$

to constrain the C blocks in equation (2.15), and


(2.18) $$\begin{array}{lcr} & \left\|\;\varrho_{\left[m,n+M\right]}-\varrho_{\left[n,m+M\right]}\;\|∼\text{Normal}(0,\epsilon \right) & \end{array}$$

to constrain the B blocks in equation (2.15). In the limit, as ϵ→0, the posterior of ϱ takes on the form given in equation (2.15). In practice, we set ϵ equal to a small constant—e.g. 0.01.

An alternative method for generating structured correlation matrices has been recently developed by Pinkney [44]; this method is more computationally efficient, but requires special initial values for Markov chain Monte Carlo (MCMC). Both methods are supported in STRAND. A general 2M×2M correlation matrix has (2M2) free parameters. The multiplex SRM, however, has a total of only (M2) free parameters in block C and (M2) + M free parameters in block B for a total of only $M^{2}$ parameters. The method in Pinkney [44] allows us to estimate only the $M^{2}$ free parameters required. The method tracks the lower and upper bounds that each element in the Cholesky factor of the correlation matrix may take for the resulting matrix to be a valid positive-definite correlation matrix with the proper block structure. The log-determinant-Jacobian of each element is added to the log-density so that the correlation matrix is uniform over the constrained structure. More details can be found in Pinkney & Ross [45].

### 2.2. Bernoulli, Gaussian and Poisson models

Bernoulli, Gaussian and Poisson variants of the model can be specified by replacing equation (2.1) with


(2.19) $$\begin{array}{lrr} & G_{\left[i,j,m\right]}∼\text{Bernoulli}\left(\text{logistic}(\theta_{\left[i,j,m\right]})\right) & \end{array}$$

or


(2.20) $$\begin{array}{lrr} & G_{\left[i,j,m\right]}∼\text{Normal}\left(\theta_{\left[i,j,m\right]},\psi \right) & \\ & \psi ∼\text{Exponential}(1.0) & \end{array}$$

or


(2.21) $$G_{\left[i,j,m\right]}∼\text{Poisson}\left(\text{exp}(\theta_{\left[i,j,m\right]})\right).$$

### 2.3. Posterior network metrics

Researchers are often interested in computing a variety of edge-level, node-level and graph-level metrics that help describe a network. These metrics, however, are typically computed directly on the observed network, which leads to each metric being a simple point estimate that is potentially sensitive to structural biases in measurement. STRAND facilities posterior estimates of these same quantities by applying the relevant calculations to each MCMC sample of the estimated latent network. See ‘Network_Metrics_Example.R’ on our GitHub for a short tutorial. This workflow is often especially important to deploy when the raw data are subject to measurement error, and the latent network can be made robust to measurement error—e.g. by accounting for individual-level censoring probability [46] or adjusting for reporter discordance [40].

### 2.4. A note on undirected networks

The basic SRM and its multiplex extension are quite general generative models for directed networks, of which undirected or symmetric networks are just a special case (where dyadic reciprocity and generalized reciprocity are maximal, which enforces the desired symmetry). There are a few technicalities involved in using the SRM on undirected networks (e.g. the same regression equation should be used for both focal and target regressions, and directed dyadic predictors should not be included in the model), and STRAND automatically checks for violations of these conditions. We provide a tutorial on our GitHub showing how to use STRAND for single-layer SRMs on undirected networks (see ‘Undirected_Example.R’). We also provide a tutorial showing how to use STRAND for a multiplex model in which at least one layer is undirected (see ‘Multiplex_Undirected_Example.R’).

### 2.5. A note on Bernoulli models

Binary outcomes are typically information-sparse relative to Poisson or Binomial outcomes. As such, parameter estimation and identification can be more challenging. Different methodologists adopt different approaches to deal with the issue. In particular, the amen package developed by Hoff [47] and the Stat-JR templates of Koster *et al.* [15,48] use a latent-variable formulation of a bivariate probit model, which estimates sender and receiver effects—but not dyadic effects—parametrically; dyadic reciprocity is then inferred indirectly by measuring correlations in residuals (see also [49]). The benefit of this approach is that it allows the three-way variance partition (between sender effects, receiver effects and dyadic effects plus residual error) to be well identified for all values of dyadic reciprocity. In contrast, STRAND estimates sender, receiver and dyadic random effects parametrically and conducts a four-way variance partition (between sender effects, receiver effects, dyadic effects and correlation-zero residual error), for both logit and probit models. When dyadic reciprocity is strong, all four variance components can be easily identified; however, when dyadic reciprocity is weak and the sample is small, the dyadic and residual error components will only be weakly identified. This is not an issue for inference using STRAND, however, since other parameters are well identified regardless (see detailed unit tests in the electronic supplementary material of [40,50]). Moreover, users can compute well-identified, amen-style, three-way variance partitions from the posterior samples returned by STRAND simply by summing the dyadic and error variance terms into a single component before standardizing each component by the sum of all variance terms. We provide a convenience function—strand_VPCs()—for processing variance partition estimates that supports both three-way and four-way partitions. In the electronic supplementary material, we show that when STRAND and amen models are fitted to the same data, their three-way variance decompositions are numerically identical (up to MCMC precision) in all cases, across a range of values of ς and ϱ.

Beyond variance partitioning, there are some other consequences of the difference in model specification worth keeping in mind. First, the interpretation of the base dyadic reciprocity estimates is different between software, like amen—which implements the classical formulation of the SRM [13, p. 143]—and STRAND—which separates potentially correlated residual error into dyadic and noise components. The definition of dyadic reciprocity in STRAND refers to dyadic correlations in the true latent social network, while the definition used in the more classic SRM formulation describes correlations in reported ties. In the special case of no reporting/measurement error, the two definitions of dyadic reciprocity are equivalent; in the presence of measurement error, however, the classic formulation of dyadic reciprocity confounds features of the true social network with features of the measurement process (i.e. noise in measurement reduces the apparent strength of dyadic reciprocity). STRAND permits specification of models that are more robust to reporting error; our model specification also remains consistent with published extensions of the SRM—e.g. for Poisson outcomes [38, p. 509], where the dyadic reciprocity estimates also reflect correlations in parametrized dyadic random effects terms. In the electronic supplementary material, we show that the bivariate probit model in amen will under-predict the *true* generative dyadic reciprocity correlation parameter, ϱ, by a factor of exactly


(2.22) $$\begin{array}{lcr} & \varkappa =\frac{\varsigma^{2}}{\varsigma^{2}+1} & \end{array}$$

*when the raw data are generated using a bivariate probit generative model with parametrized sender, receiver and dyadic random effects* (where the generative dyadic effects have variance $\varsigma^{2}$ and correlation ϱ, and the uncorrelated measurement error has unit variance) (see Snijders & Kenny [49] for an earlier proof of the same scaling term in equation (2.22)). This is not to say that the estimates of amen are biased or incorrect, only that they represent correlations in a *different quantity*—i.e. of observed/measured outcomes—than the unadjusted estimates returned by STRAND—which represent correlations in the unobserved latent network of social ties.

Additionally, the posterior uncertainty in estimates of ϱ returned by STRAND is normally much larger than what is found in estimates of ϱ returned by amen. When the outcome data do not show strong signs of dyadic correlations, STRAND must integrate over the possible parameter states that would explain such data. Such parameter states might include (i) a low frequency of dyadic ties in the true latent social network or (ii) a high frequency of dyadic ties in the true latent social network coupled with a high level of measurement error/noise in reporting. Ready & Power [51] show that human self-report networks are often characterized by substantial levels of discordance (of the order of 90%) between members of a dyad reporting on the same binary tie, as such STRAND is designed to be conservative and not assume that the level of correlation in reported outcomes maps identically onto correlations in the true latent network. STRAND users preferring to report measurement-level dyadic reciprocity estimates comparable with those of amen and similar packages can compute the variance-adjusted correlation, $\varrho \frac{\varsigma^{2}}{\varsigma^{2}+1}$, post-sampling. This involves simply changing the argument: mode=‘cor’ in the strand_VPCs() function to: mode=‘adj’ (see electronic supplementary material).

When fitting STRAND models to Bernoulli data, we caution against directly interpreting the raw variance terms marginally. We recommend computing variance partition coefficients (VPCs) and considering the three-way partitioning and the variance-adjusted correlations, as well as the unadjusted estimates. It is always good to check fitted models using the workflows discussed in Gelman *et al.* [52] and McElreath [39]: notably, check $\hat{r}$ values, effective number of posterior samples, trace plots, pairs plots and posterior predictive distributions.

## 3. STRAND for multiplex analysis

To fit multiplex network analysis models using simple base-R syntax, we provide an R package called STRAND, which builds and runs Bayesian models using Stan [53,54], while requiring users to know only basic lm-style model specification.

Since STRAND depends on Stan [54] and CmdStanR [53], users must install these programs prior to installing STRAND. We provide quick-start links on the package’s GitHub homepage: https://github.com/ctross/STRAND. Installation and loading of STRAND are then simple: just run three lines of code from R:

**Figure:**
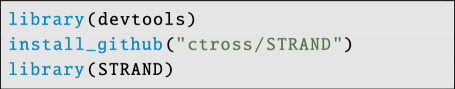


All of the example code presented below can also be found in the Tutorials section of the package’s GitHub: https://github.com/ctross/STRAND/tree/main/tutorials.

### 3.1. Simulating multiplex network data

An essential part of model specification involves testing that an analytical model can recover the parameters used to create simulated data. As such, STRAND provides both data simulation engines and Bayesian analysis models. We begin our tutorial by first showing how users can simulate multiplex network data and then showing how to analyse empirical data. While it is tempting to skip ahead, we recommend always testing analysis pipelines using simulated data.

To simulate a multiplex network, we first create a simulated relational database. The synthetic data might include individual measures like ‘body mass’, dyadic measures like ‘relatedness’ and block-structuring variables like ‘social-group’, where the probability of network ties in each output layer depends on the block/group identity of both sender and receiver. Alternatively, users may wish to simulate network data that are not influenced by covariates. Either way, the simulate_multiplex_network function is used to deploy the simulation engine:

**Figure:**
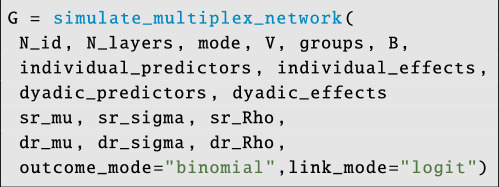


The input arguments are fairly intuitive: N_id gives the number of individuals/nodes in the network, N_layers gives the number of network layers to be created, outcome_mode ∈{‘bernoulli’, ‘binomial’, ‘poisson’} indicates the type of output model to use when generating edges and link_mode ‘logit’, ‘probit’, ‘log’ gives the link function to use. The argument V gives the number of blocking variables to use in each layer, groups is an N_id by V data frame of factor variables that create group structure in each network layer and B is a length-N_layers list of length-V lists of matrices giving the between block tie probabilities for each blocking variable in each layer (see electronic supplementary material, GitHub tutorial for a commented example).

Next, the argument individual_predictors is an N_id by N_ind_vars data frame of individual-level predictor variables that may influence ties in each network layer. The argument individual_effects is an N_layers long list of matrices of dimension 2 by N_ind_vars. The first row of each matrix gives the effects of each individual predictor on nodal out-degree (or out-strength), and the second row gives the effects of each individual predictor on nodal in-degree (or in-strength). The argument dyadic_predictors is an N_id by N_id by N_dyadic_vars array of dyad-level predictor variables that may influence ties in each network layer. The argument dyadic_effects is a length-N_layers list of coefficient vectors of length N_dyadic_vars.

Finally, we have the parameters that control random effects within and between network layers. The argument sr_mu is a vector of length 2N_layers and gives the average effect size for sender and receiver random effects in each layer; in most cases, this should be a vector of zeros. The argument sr_sigma is a vector of length 2N_layers and controls the variance in sender and receiver random effects for each layer; the first N_layers parameters control the variance of sender effects in each layer, and the next N_layers parameters control the variance of receiver effects in each layer. The argument sr_Rho must be a valid, positive-definite correlation matrix of dimension 2N_layers by 2N_layers; this matrix controls ‘generalized reciprocity’, e.g. it controls the extent to which individual i’s tendency to send ties (to anyone) in network layer l correlates with individual i’s tendency to receive ties (from anyone) in network layer m. The argument dr_mu is a vector of length N_layers and gives the average effect size for dyadic random effects in each layer; in most cases, this should be a vector of zeros. The argument dr_sigma is a vector of length N_layers and controls the variance in dyadic random effects for each layer. The argument dr_Rho must be a valid, positive-definite correlation matrix of dimension 2N_layers by 2N_layers; this matrix controls ‘dyadic reciprocity’, e.g. it controls the extent to which individual i’s tendency to send ties (to j) in network layer l correlates with individual i’s tendency to receive ties (from j) in network layer m. Note that this matrix should have the block structure described in equation (2.15).

The output object G will be a list containing a multiplex adjacency array called network (of dimension N_layers by N_id by N_id), along with other relevant data, including an exposure variable (of the same dimensionality) if the Binomial outcome mode is selected.

The simulation of multiplex networks requires a large number of parameters. Creating good simulations can therefore be a somewhat daunting task. As such, on our GitHub page, we provide a more in-depth, commented-code tutorial, where we show how to simulate covariate data, define parameters and organize them for STRAND (see ‘Multiplex_Binomial_Simulation_Example.R’).

## 4. Modelling multiplex networks

### 4.1. Building data objects

The first step in modelling empirical data is to organize all of one’s data types into a standardized format that can be processed automatically in STRAND. Social network data are normally complex, with some variables being reported at the level of the individual and others being reported at the level of the dyad. The make_strand_data function serves to organize all of these data into a unified format that can be read by later functions. After data are compiled, they can then be analysed with simple, lm-style function calls, as we discuss below.

To begin, we will use data (n=93 individuals) from a set of network-structured economic games (i.e. the RICH games introduced by Gervais [55]) collected in a field site in rural Colombia [56,57]. In this dataset, there are three layers of Bernoulli outcome networks: the first layer describes if individual i gave coins to individual j in a giving game, the second layer describes if individual i took coins from individual j in a taking game and the third layer describes if individual i paid money to reduce the payout of individual j in a costly punishment game.

With such data, we can pose research questions like: (i) Are people who are more generous in the giving game less likely to be targets of exploitation in the taking game and punishment in the costly reduction game? (ii) Are giving behaviour, taking behaviour and costly reduction behaviour reciprocal at a dyadic level, within and across network layers? (iii) Are covariates like age, sex, ethnicity, wealth and food insecurity related to behaviour in each economic game?

To address such questions, we first store each outcome layer in a labelled list:

**Figure:**
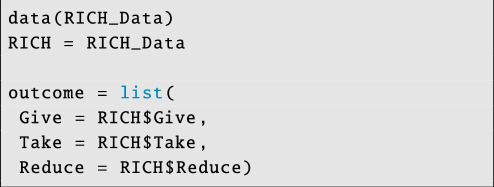


Note that outcome data should be Gaussian, Bernoulli, Binomial or Poisson. In the current version of STRAND, all layers in the multiplex must also be of the same distributional type.

Next, we store dyadic predictor variables in a labelled list:

**Figure:**
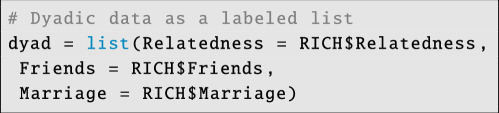


Dyadic covariate data can include numeric variables, indicator variables or even categorical variables. These dyadic covariate data can be used to estimate associations between dyad-level characteristics—such as genetic relatedness or physical proximity—and the likeliness of a tie in each of the outcome networks.

We store individual-level data in a data frame:

**Figure:**
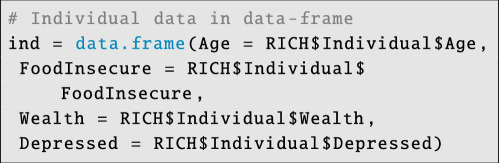


The individual-level data can include numeric variables, indicator variables or categorical variables. These data can be used to estimate associations between individual-level characteristics and the likeliness of either sending or receiving a tie in each of the outcome layers.

Finally, we store the individual-level data used to create block structure in a separate data frame:

**Figure:**
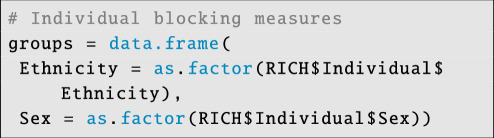


Although block-structuring variables are also individual-level data, they are treated differently from other variables by STRAND; these variables must be factors and are used to create random intercept offsets unique to the interaction of sender and receiver block IDs.

With all of the data organized, we can build the final STRAND data object:

**Figure:**
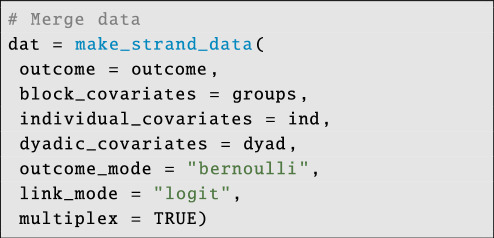


The first four arguments make_strand_data retrieve the relevant data objects. The outcome_mode argument takes values ∈{‘gaussian’, ‘bernoulli’, ‘binomial’, ‘poisson’}. The ‘gaussian’ option is for normally distributed data, the ‘bernoulli’ option is for binary tie data (e.g. for self-report/name-generator data), the ‘poisson’ option is for raw count data (e.g. the number of times GPS trackers were within 5 m of each other) and finally the ‘binomial’ option is for proportion data. If the outcome mode is set to ‘binomial’, then an exposure variable must also be provided. The exposure variable is a labelled list containing matrices of sample sizes—i.e. counts of the number of times that each dyadic tie could have been observed in each outcome layer. Since our outcome networks in this example are binary indicators, we select outcome_mode = ‘bernoulli’ and link_mode = ‘logit’. Some users prefer probit models, and can set: link_mode = ‘probit’. Finally, the argument multiplex = TRUE instructs STRAND to structure the data for a multiplex model.

### 4.2. Fitting the basic multiplex model

Once the data are organized, we can deploy the Bayesian model using the fit_multiplex_model command. For this example, we will actually fit two models. In the first model, we will omit covariates, using the lm-syntax ∼1 to indicate that each sub-model is ‘intercept only’:

**Figure:**
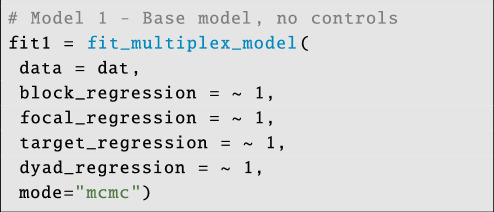


Running this command will start model compilation and then MCMC sampling. Users should be aware that Bayesian models fitted via MCMC can take a long time to run and are memory-intensive. This is especially true of highly parametrized models like the multiplex SRM. An old adage, however, is that we often spend months or years collecting data, and so we should be happy waiting a few hours to days for our models to fit. The parameter complexity of the multiplex model used here scales at roughly N_layers × N_id${,}^{2}$, and so a four-layer model with 250 individuals requires more than a quarter-million parameters! As such, STRAND models are generally best suited to cases where the sample is not too large (e.g. ⪅ 400 individuals), and/or the number of network layers is small (e.g. ⪅ 16 layers).

STRAND provides two convenience functions for processing and summarizing samples. To compute VPCs and, if desired, amen-style, variance-adjusted correlation terms, users can run:

**Figure:**
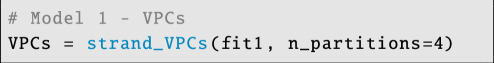


For VPCs analogous to those of amen, one can set n_partitions = 3 to conduct a three-way partition of variance between focal, target and residual terms in each layer. Additionally, STRAND permits a four-way partition of variance between focal, target, dyadic and residual terms. In Bernoulli models, when the estimated dyadic reciprocity within a layer is low, the posterior uncertainty in the partition between dyadic and error terms is likely to be large. Users can set mode = ‘adj’ to get variance-adjusted correlations (e.g. see equation (2.22)) or mode = ‘cor’ to get unadjusted correlations.

All other parameters can be processed using a specialized summary function:

**Figure:**
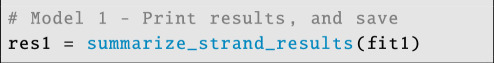


This function prints and saves summary statistics—like the posterior median and highest posterior density interval—for each parameter in the model in tabular form (e.g. see tables 1 and 2).

**Table 1.** Coefficient estimates from the giving game in the Colombian dataset [57]. Effects are presented as posterior medians with 90% credible intervals. Note that ‘block’ estimates cannot be interpreted marginally. Users must compute contrast coefficients between categories to check if observed differences between categories are statistically reliable.

category	outcome	effect	median	5% CI	95% CI
block	give	intercept	1.15	−1.22	3.81
block	give	Afro-to-Afro	−3.11	−5.03	−1.25
block	give	Afro-to-Emb	−4.46	−6.31	−2.48
block	give	Emb-to-Afro	−4.83	−6.79	−2.89
block	give	Emb-to-Emb	−2.36	−4.4	−0.39
block	give	fem-to-fem	−3.81	−5.68	−1.83
block	give	fem-to-male	−4.53	−6.48	−2.67
block	give	male-to-fem	−3.89	−5.91	−2.11
block	give	male-to-male	−3.46	−5.41	−1.58
focal	give	age	−1.1	−2.09	0.1
focal	give	log wealth	0.01	−0.34	0.45
focal	give	food insecure	0.15	−0.39	0.65
focal	give	depressed	−0.11	−1.06	0.72
target	give	age	1.68	0.69	2.65
target	give	log wealth	−0.24	−0.55	0.11
target	give	food insecure	−0.01	−0.44	0.42
target	give	depressed	0.14	−0.55	0.74
dyadic	give	relatedness	3.76	3.06	4.32
dyadic	give	friends	2.11	1.46	2.65
dyadic	give	marriage	4.33	3.47	5.04
sigma	give	focal	1.24	1	1.53
sigma	give	target	0.9	0.7	1.11
sigma	give	dyadic	1.86	1.48	2.26

**Table 2.** Generalized and dyadic reciprocity estimates from model 2 fitted to the Colombian data [57]. Effects are presented as posterior medians with 90% credible intervals. Note that the correlation estimates shown here are between the two columns labelled ‘outcome’ in each row. In the ‘generalized’ section, estimates give correlations between random effects affecting in-degree (in) and out-degree (out). In the ‘dyadic’ section, estimates give correlations between random effects affecting i-to-j flows and j-to-i flows.

category	outcome	outcome	median	5% CI	95% CI
generalized	give (in)	reduce (out)	0.36	0.12	0.57
generalized	give (in)	reduce (in)	−0.07	−0.4	0.23
generalized	give (in)	take (in)	−0.36	−0.57	−0.15
generalized	give (out)	reduce (out)	0.17	−0.06	0.41
generalized	give (out)	give (in)	0.07	−0.17	0.32
generalized	give (out)	reduce (in)	0.06	−0.23	0.36
generalized	give (out)	take (out)	−0.09	−0.31	0.1
generalized	give (out)	take (in)	−0.11	−0.29	0.14
generalized	reduce (out)	reduce (in)	−0.16	−0.5	0.17
generalized	take (in)	reduce (out)	−0.09	−0.3	0.14
generalized	take (in)	reduce (in)	0.67	0.47	0.86
generalized	take (out)	reduce (out)	0.22	−0.01	0.43
generalized	take (out)	give (in)	0.14	−0.07	0.34
generalized	take (out)	take (in)	−0.03	−0.2	0.16
generalized	take (out)	reduce (in)	−0.17	−0.44	0.13
dyadic	give (i to j)	give (j to i)	0.56	0.39	0.75
dyadic	take (i to j)	take (j to i)	0.01	−0.12	0.12
dyadic	reduce (i to j)	reduce (j to i)	−0.03	−0.37	0.33
dyadic	give (i to j)	reduce (i to j)	−0.36	−0.58	−0.17
dyadic	give (i to j)	take (i to j)	−0.63	−0.74	−0.48
dyadic	take (i to j)	reduce (i to j)	0.61	0.48	0.73
dyadic	give (i to j)	reduce (j to i)	−0.06	−0.29	0.12
dyadic	give (i to j)	take (j to i)	−0.3	−0.43	−0.18
dyadic	take (i to j)	reduce (j to i)	0.1	−0.09	0.23

**Figure 2.**
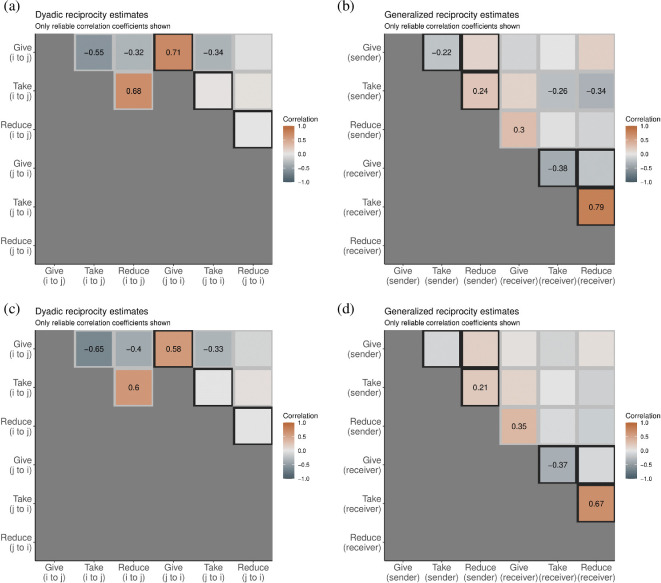
Dyadic and generalized reciprocity correlations, within and between layers, from a coastal Colombian community [57]. Frames (a) and (b) are from model 1, without controls. Frames (c) and (d) are from model 2, with controls. Positive correlations are orange, and negative ones are blue. Only parameters with a 90% credible interval that do not intersect zero are plotted numerically. We see strong correlations in experimental economic behaviour at both dyadic and generalized levels. Such effects are mostly robust to the set of included controls.

Note, however, that because STRAND returns all coefficient estimates for each network layer, displaying and interpreting coefficients in tabular form is often tiresome. It is generally better to visualize the coefficient estimates in a plot. We provide two convenience functions for visualizing results in this way. The first function, called multiplex_plot, visualizes the dyadic and generalized reciprocity matrices as correlation heatmaps (figure 2):

**Figure:**
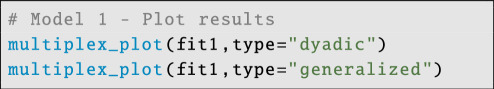


There are three main arguments to this function: type ∈{‘dyadic’, ‘generalized’} indicates which matrix to plot, HPDI ∈(0,1) indicates which values get labelled (by default the 90% interval is calculated, and only effects that are reliably non-zero are labelled) and finally the Boolean variable export_as_table indicates if the tabular data should be exported so that users can make the plot externally using their own ggplot settings.

**Figure 3.**
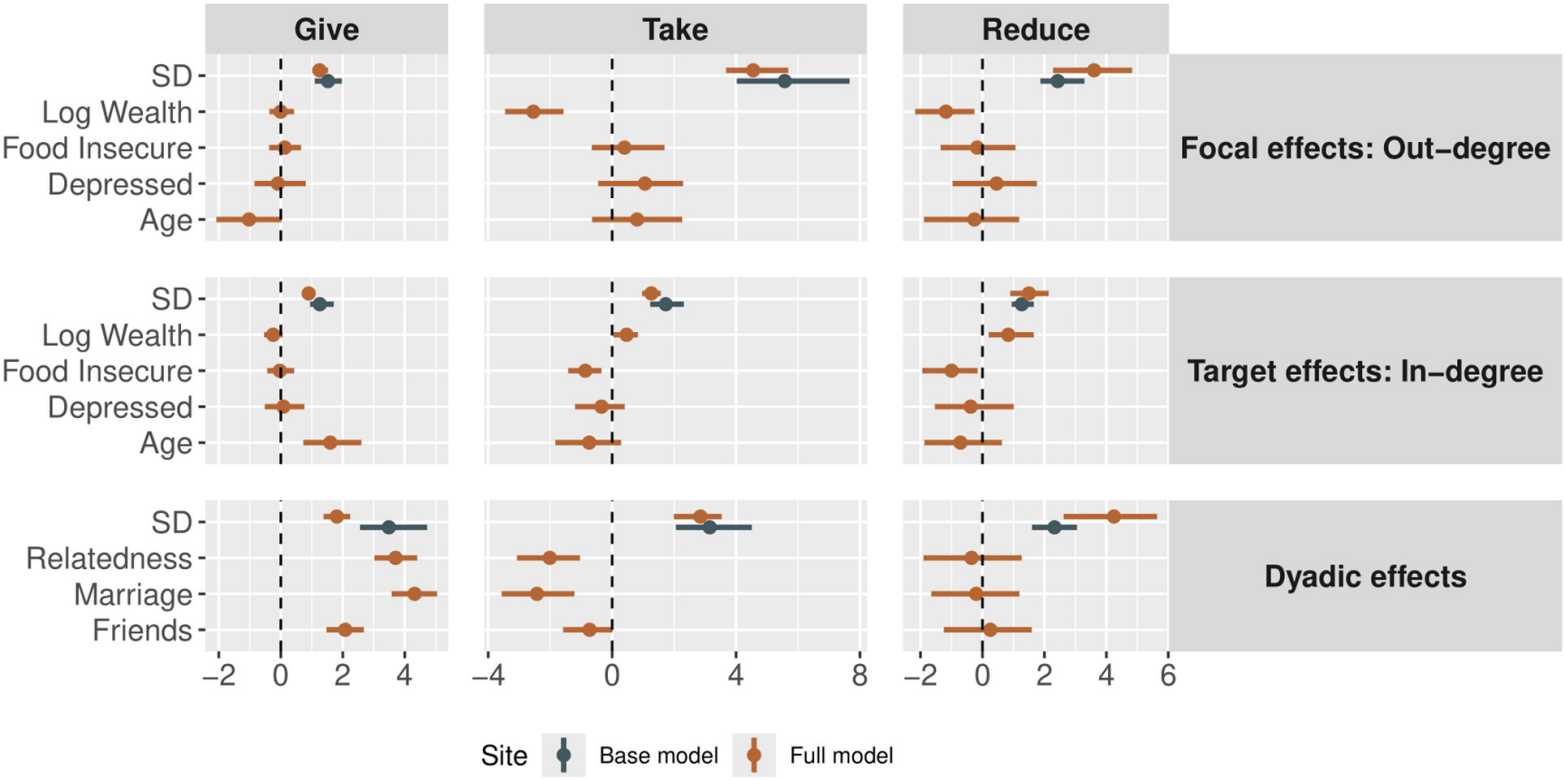
Parameter estimates for the effects of various covariates on the structure of network ties in each layer of the Colombian dataset [57]. Points represent the posterior medians, and bars represent the 90% highest posterior density intervals. Blue points show the parameters estimated in model 1, and orange points show the parameters estimated in model 2. The parameters labelled ‘SD’ are the standard deviations of the random effects. All other estimates are slope coefficients. Dyadic variables like friendship, marriage and relatedness are the strongest predictors of experimental economic behaviour, but age, wealth and food security have influences on node-level out-flows and in-flows as well.

The second plot visualizes model parameters using a ‘caterpillar’ or ‘forest’ plot, which displays the effect sizes and credible regions relative to a dashed vertical line at zero, which represents ‘no effect’ (figure 3):

**Figure:**
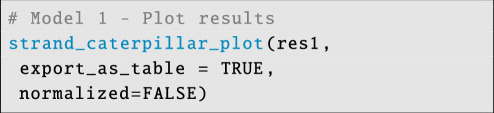


There are three main arguments to this function: submodels indicates which effects to plot, the variable export_as_table indicates if the tabular data should be exported so that users can apply their own ggplot settings and the Boolean variable normalized indicates if the posteriors should be normalized by dividing by the width of the highest posterior density interval.

### 4.3. Priors

By default, STRAND uses weak priors for most parameters, so that the likelihood mainly determines the posterior. Weakly regularizing unit-normal priors, however, are used on slope coefficients to prevent over-fitting in small samples [39]. In nearly all cases, users will not need to modify the default priors. We do, however, recommend standardizing all predictor variables prior to model-fitting so that the priors on the slopes are of consistent strength across predictors. We provide a function make_priors(), which lets users change priors as needed. See Ross *et al.* [50] for additional details.

### 4.4. Controlling for covariates

In the second model, we will add predictors at the block, individual and dyadic level. STRAND uses lm-syntax—e.g. ∼ Var1 + Var2—to define regression equations. As this is a multiplex model, STRAND will deploy the same regression equations to each network layer. In total, four different regression equations must be specified in the model call:

**Figure:**
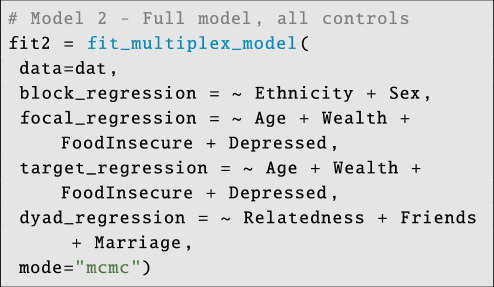


In the first equation, we estimate block-level effects for ethnicity and sex using the argument: block_regression = ∼Ethnicity + Sex. These effects are indicative of how the likeliness of ties varies as a function of the interaction of block categories—i.e. we can test if Afrocolombian-to-Emberá ties are more or less likely than Afrocolombian-to-Afrocolombian ties, Emberá-to-Afrocolombian ties or Emberá-to-Emberá ties; likewise, with male-to-female ties relative to male-to-male, female-to-male or female-to-female ties. It should be noted that individual-level categorical variables like ethnicity or sex could instead be supplied as focal or target predictors if the interaction between category levels is not of interest. However, the same variable should not be included as both a block predictor and a focal/target predictor, as this will lead to an underidentified model.

Next, the focal regression model, focal_regression = ∼Age + Wealth + FoodInsecure + Depressed, explores how individual-level features are related to the propensity to send outgoing ties in each layer (i.e. it measures the effects of individual-level covariates on *out-degree* or *out-strength*). Similarly, the target regression model, target_regression = ∼Age + Wealth + FoodInsecure + Depressed, explores how individual-level features are related to the propensity to receive incoming ties (i.e. it measures the effects of individual-level covariates on *in-degree* or *in-strength*). The dyad regression model, dyad_regression = ∼Relatedness + Friends + Marriage, explores how the likeliness of directed dyadic ties in each network layer is associated with relational features of a dyad, like whether two individuals are genetically related, friends or marriage partners.

### 4.5. Interpreting results

#### 4.5.1. Variance partitions

**Figure 4.**
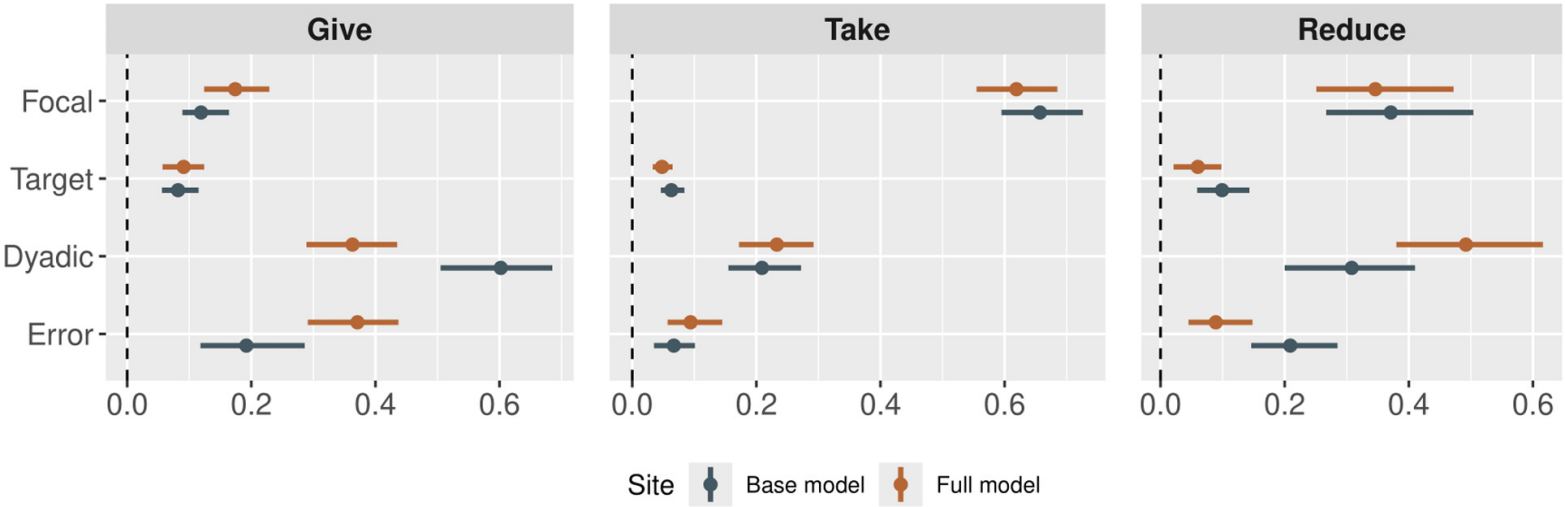
Estimates of the VPCs (on the latent scale) for each layer of the Colombian dataset [57]. Points represent the posterior medians, and bars represent the 90% highest posterior density intervals. Blue points show the parameters estimated in model 1, and orange points show the parameters estimated in model 2. Giving propensity appears mostly influenced by dyadic random effects, while taking propensity is mostly explained by focal random effects.

Figure 4 shows the VPCs for each layer. These estimates are indicative of the fraction of variance (on the latent scale) that is explained by each of the different random effects. Some users make calculation of such coefficients a major part of their workflows [58]; others, notably McElreath [39], advocate against interpreting parameters from nonlinear models on transformed scales and, instead, recommend computing the implications of differing counterfactuals by simulating outcomes from the posterior. We see merit in both approaches, and so STRAND returns a results object that facilitates both kinds of calculations.

#### 4.5.2. Dyadic reciprocity

Figure 2 shows the dyadic and generalized reciprocity matrices as correlation heatmaps. In figure 2a, we see dyadic reciprocity estimates—within and between layers—under the first model, with no controls. There are two ‘triangles’ of effects highlighted in light grey and a ‘diagonal slash’ of effects highlighted in dark grey. The left-most triangle shows correlations in random effects ‘within the mind of i’. That is, it shows correlations in the way i acts on j across network layers. In our case, if i gives to j, then i is unlikely to take from or reduce j, but if i takes from j, then i is more likely to pay to reduce j in the costly reduction game.

The diagonal slash of effects shows dyadic reciprocity within layers. Here, we see that if individual i gave to individual j, that individual j is much more likely than chance to give to individual i. We do not find evidence of such dyadic reciprocation in the taking or reduction games.

The right-most triangle shows correlations in random effects ‘between i and j, between layers’. That is, it shows correlations in the way i acts on j in layer l with the way j acts on i in layer m. In our case, if i gives to j in the giving game, then j is less likely to take from i in the taking game.

In figure 2c, we see dyadic reciprocity estimates under the second model, with controls. Even though relatedness, marriage and friendship are important dyadic drivers of transfers in the economic games (figure 3), we find that all of our key dyadic reciprocity estimates remain reliable when controlling for the full set of controls in each layer.

#### 4.5.3. Generalized reciprocity

In figure 2b, we see generalized reciprocity estimates—within and between layers—under the first model, with no controls. There are two ‘triangles’ of effects highlighted in dark grey and a ‘square’ of effects highlighted in light grey. The upper-left triangle shows correlations in the random effects controlling each individual’s propensity to emit ties in each layer (e.g. their out-degree propensity). In our case, we see that if i gives many coins to others in the giving game, then i is less likely to take from many others in the taking game. However, we see that if i takes many coins from others in the taking game, then i is also more likely to pay to reduce many others in the costly reduction game. This is consistent with both exploitation and costly punishment being used as levelling mechanisms [59].

The lower-right triangle shows correlations in the random effects controlling each individual’s propensity to receive ties in each layer (e.g. their in-degree propensity). In our case, we see that if i receives many coins from others in the giving game, then i is less likely to be taken from in the taking game. However, we see that if i is frequently taken from in the taking game, then i is also likely to be reduced by others in the costly punishment game.

Lastly, the grey square of effects shows correlations across layers and across modes (out-degree versus in-degree). Interestingly, we find that individuals who pay a personal cost to reduce others are actually preferential targets of giving, in general, by others. Finally, individuals who take coins from many others in the taking game are themselves preferential targets of both taking and costly reduction. In figure 2d, we see that, in contrast to what we saw with dyadic reciprocity, a few of the generalized reciprocity estimates cease to remain reliable after accounting for the full set of controls.

#### 4.5.4. Individual and dyadic covariate effects

Covariate effects are best represented using forest plots, as we show in figure 3. In terms of predicting out-degree, we find that older people are less likely to give to others in the giving game and that wealthier individuals are less likely to take from or pay money to reduce others. In terms of predicting in-degree, we find that older people are more likely to be targets of giving in the giving game, while wealthier individuals are less likely to be targets of giving. Wealthy individuals are more likely to be targets of taking and costly reduction, and individuals who reported food insecurity are less likely to be targets of taking or costly reduction. Finally, at a dyadic level, relatedness, marriage and friendship are positively predictive of giving ties and negatively predictive of taking ties.

#### 4.5.5. Block effects

At first glance, it is common for the posterior distributions of block effects to look like they overlap when plotted using forest plots, even if there are statistical differences between effects. This phenomenon arises because block models are typically underidentified—i.e. a small increment, υ, can be added to the intercept and subtracted from every block offset, without changing the likelihood. In a Bayesian framework, the model is still well specified because even weak priors identify the model, but care must be taken to correctly interpret block estimates. As such, researchers must be sure to calculate posterior contrasts (see [39, §5]) for any block effects they may be interested in.

For example, in table 1, it appears that the Afrocolombian-to-Afrocolombian offset is −3.11 (−5.03, −1.25) and that the Afrocolombian-to-Emberá offset is −4.46 (−6.31, −2.48). These estimates appear to overlap substantially; however, if we calculate the contrast coefficient: 1.33 (0.55, 2.09), we find that Afrocolombian-to-Afrocolombian ties are reliably more likely than Afrocolombian-to-Emberá ties.

To compute contrasts, STRAND provides a function called process_block_parameters; users can supply the focal and ‘base-case’ variable names to get the posterior difference (e.g. in log-odds) between the two categories of interest:

**Figure:**
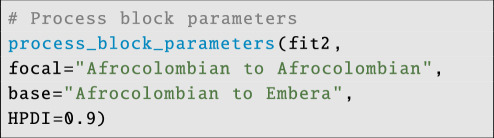


## 5. Modelling animal networks

To demonstrate how to fit a multiplex model to animal social networks, where outcomes are typically binomially distributed, we draw on behavioural data from captive Guinea baboons published by Gelardi [60,61]. These network data consist of counts of grooming, presenting (defined as: ‘approaching another individual gently with or without lipsmacks and grunts and presenting the rear’ [61]) and threatening behaviours between n=19 individuals, paired with exposure data on the number of scans in which such ties could be observed.

We investigate three questions: (i) Does the probability of individual i grooming individual j correlate with the probability that individual j grooms individual i? (ii) Is the probability of individual i grooming individual j associated with whether individual j ‘presents’ to individual i (i.e. is there dyadic reciprocity across layers)? (iii) Are age and sex predictive of behaviour in any of the three considered network layers?

As before, we start by organizing the outcome data:

**Figure:**
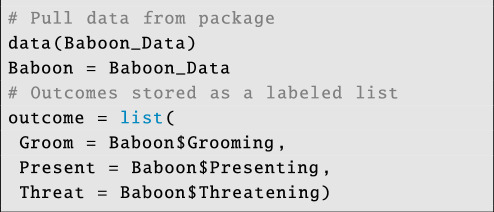


Now, we have to provide exposure data, since the outcome data are binomial:

**Figure:**
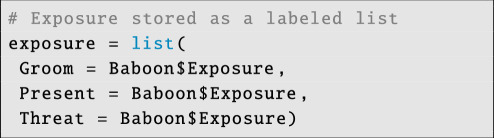


The exposure variable here is the same for each network layer. However, in most cases, users will need to provide unique exposure matrices for the relevant outcome matrices. Note that the names of exposure and outcome variables must match, as must the order in which they are declared.

Individual-level data are stored as a data frame:

**Figure:**
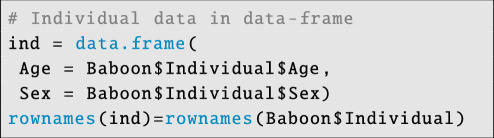


Now we merge the data files:

**Figure:**
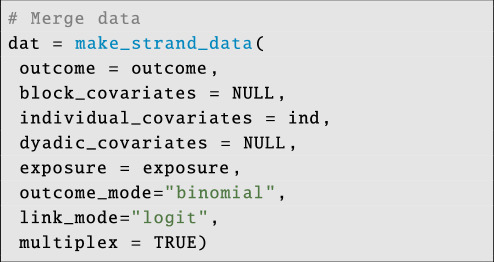


Note that since we omit block and dyadic covariates, we set their values to NULL. We add the call to integrate dyadic sample size/exposure data by writing: exposure = exposure, and we set the outcome mode for our Binomial model with: outcome_mode = ‘binomial’. After this, the model is called:

**Figure:**
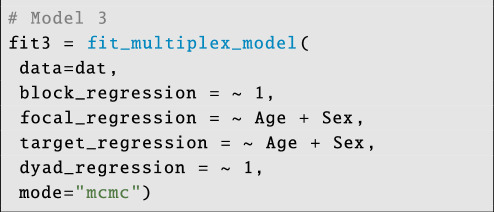


The results can then be visualized using the tools we introduced earlier.

### 5.1. Interpreting results

#### 5.1.1. Dyadic reciprocity

**Figure 5.**
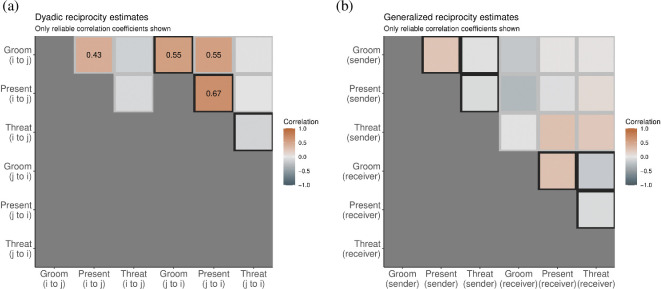
Dyadic (a) and generalized (b) reciprocity estimates, within and between layers, from a population of captive Guinea baboons [60,61]. Positive correlations are orange, and negative ones are blue. Only parameters with a 90% credible interval that do not intersect zero are plotted numerically. We see strong correlations within and between grooming and presenting layers at the dyadic level, but no strong correlations at the generalized level.

Figure 5 shows dyadic and generalized reciprocity matrices as correlation heatmaps. In figure 5a, we see dyadic reciprocity estimates—within and between layers. We note that, if i grooms j, then i is more likely to present to j. Similarly, if i grooms j, then j is more likely to present to i. These correlations arise in large part because—on the diagonal slash highlighted in dark grey—both grooming and presenting show strong signs of dyadic reciprocity within layers.

There is little evidence that threatening behaviour is related to dyadic behaviour in the other two network layers, largely because the count of such events in the raw data is small, with threatening events occurring at only 5% the frequency of grooming events, and 10% the frequency of presenting events. This lack of evidence will be reflected in wide posterior distributions. Users should always investigate if apparent null effects are supported by evidence (e.g. in the case of narrow posteriors that exclude the plausibility of substantial effects) or driven by a lack of statistical power (e.g. in the case of wide posteriors).

#### 5.1.2. Generalized reciprocity

In figure 5b, we see generalized reciprocity estimates—within and between layers. It turns out that no effects are reliable at the 5% level. This means that, after accounting for dyadic random effects and fixed predictors for age and sex, there are no statistically reliable associations between any of the nodal random effects vectors.

#### 5.1.3. Individual covariate effects

**Figure 6.**
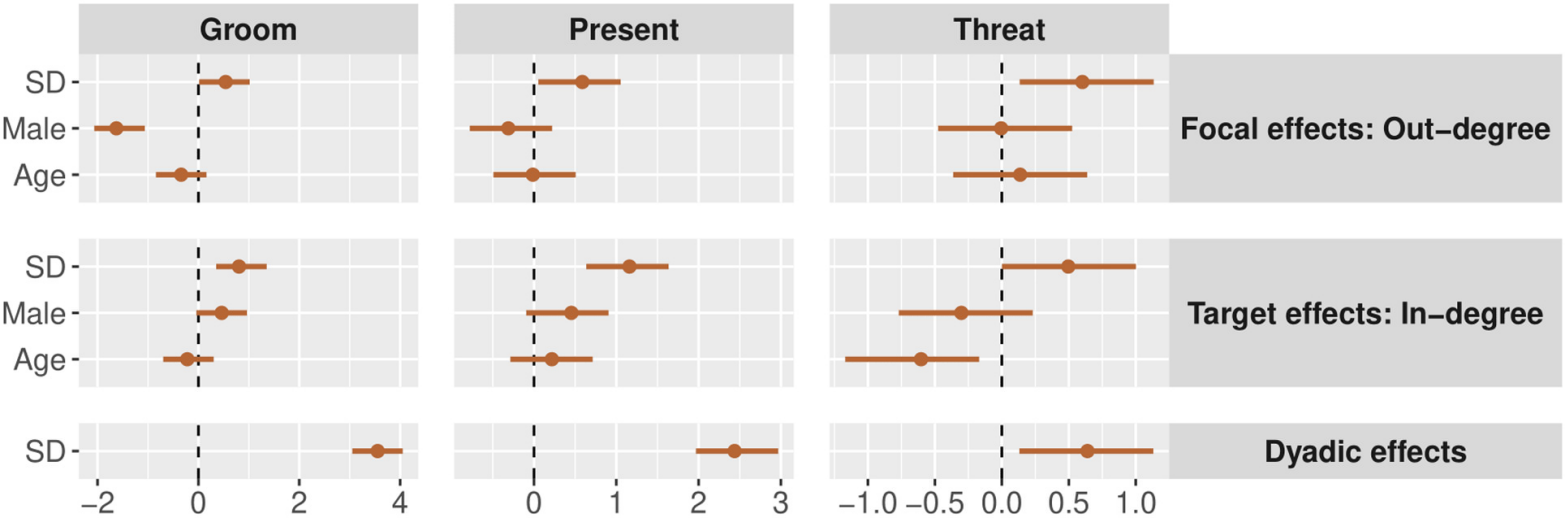
Parameter estimates for the effects of covariates on the structure of network ties in each layer of the baboon network data [60,61]. Points represent the posterior medians, and bars represent the 90% highest posterior density intervals. The parameters labelled ‘SD’ are the standard deviations of the random effects. All other estimates are slope coefficients. We observe only limited effects of sex and age on node-level properties: males groom others less frequently, and older individuals are less likely to be threatened by others.

In figure 6, we plot the effects of age and sex on tie probability in each network layer. We find that males are less likely to groom others in general, but are more likely to be groomed by others. Males were more likely to be presented to, but were no more likely to present to others. Lastly, there was no effect of sex on threatening behaviour, in either direction. Age only had an impact on a single variable: older individuals are less likely to be on the receiving side of threat displays.

## 6. Model validation

In order to ensure the quality of the software, we conduct a suite of tests. Initial validation of the SRM model structure and covariate processing pipelines was performed over large parameter sweeps [40,50]. Here, we add a new set of tests to ensure that we can recover correct dyadic and generalized reciprocity estimates, within and between network layers, for a range of multiplex models with differing numbers of layers and nodes. Then, we conduct a simple parameter recovery check of the multiplex model, by first simulating a single three-layer multiplex network—with a full set of focal, target, dyadic and block predictors—and then recovering the parameters used to simulate the data.

### 6.1. Testing dyadic and generalized reciprocity estimates

Here, we first use the simulation engine to create 70 artificial datasets with sample sizes ranging between 10 and 145 individuals and number of network layers ranging between 2 and 8 (and we repeat this process for Binomial, Poisson and even Bernoulli outcome modes). We then fit each dataset using the relevant analytical model and compare the true and estimated reciprocity matrices using the Frobenius norm, a measure of distance between matrices [62]. More specifically, we take the true generative correlation matrix, ρ, and samples from the posterior correlation matrix, $\hat{\rho }$, and we evaluate:


(6.1) $$F(\rho ,\hat{\rho })=\sqrt{Tr\left(\left(\rho -\hat{\rho }{)}^{T}(\rho -\hat{\rho }\right)\right)}.$$

This lets us build up a posterior distribution of the divergence between ρ and $\hat{\rho }$. As all elements of $\hat{\rho }$ approach the corresponding elements of ρ, the divergence given by $F(\rho ,\hat{\rho })$ tends to zero. In order to assess the expected Frobenius norm between ρ and a random matrix of a given dimensionality, we sample a correlation matrix from the prior, $\dot{\rho }$, and calculate $F(\rho ,\dot{\rho })$. We iterate this process by simulating 500 random samples of $\dot{\rho }$ from the prior and building up a distribution of the Frobenius norm between ρ and random correlation matrices.

**Figure 7.**
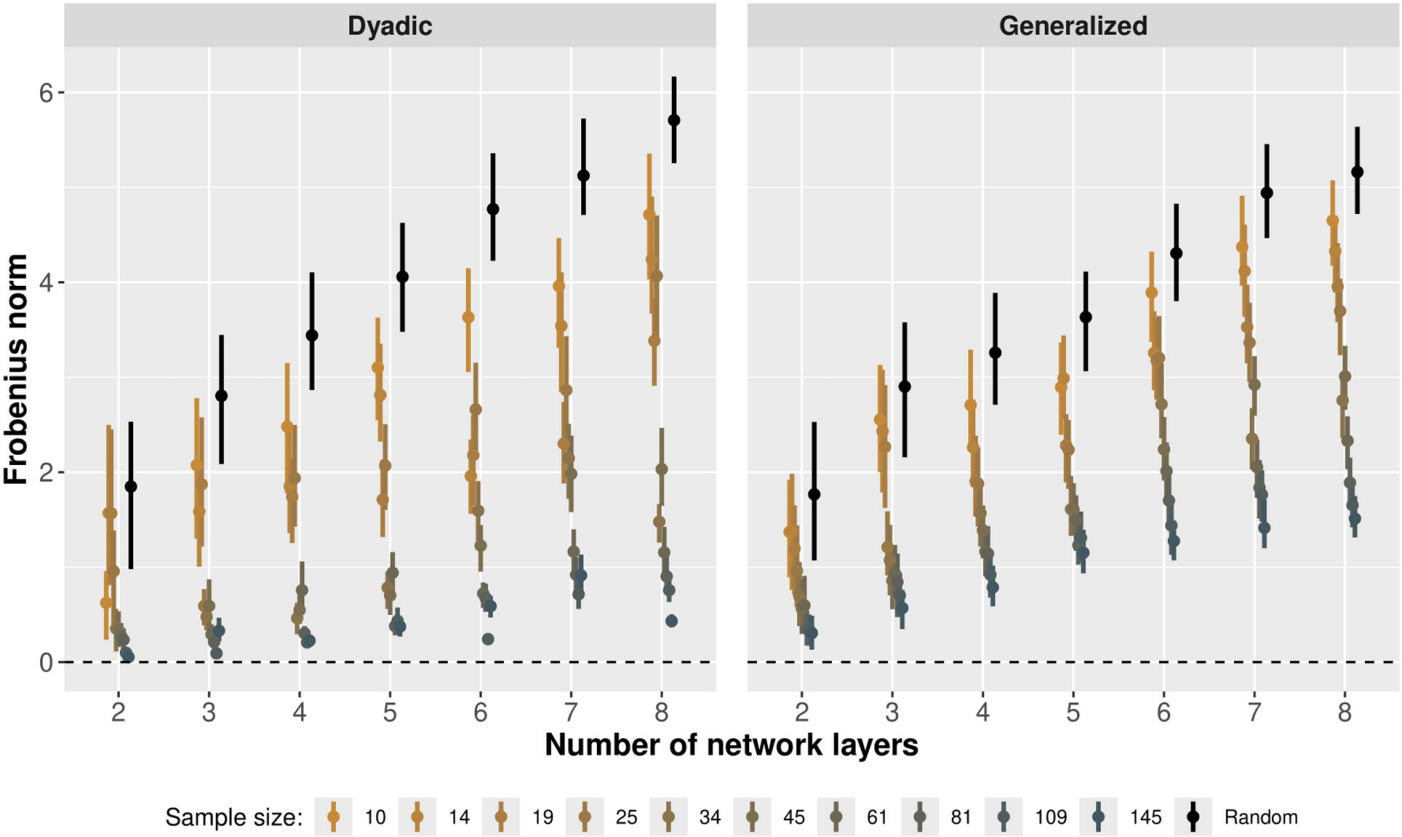
Frobenius norm between generative and posterior or random correlation matrices (Binomial model). Black points represent the median Frobenius norm between generative and random correlation matrices; bars represent the 90% highest posterior density intervals. Coloured points represent the Frobenius norm between generative and posterior correlation matrices. We plot the same estimates for various sample sizes, as indicated by colour shifting from orange (small samples) to blue (large samples). In general, we find that our posterior correlation matrices are substantially closer to the true generative correlation matrix than random correlation matrices under the prior, especially as sample size increases. This indicates that our model is accurately recovering the parameters of the generative model.

In figure 7, we plot the results of our simulation study for Binomial outcomes. The black distributions plot the Frobenius norm between ρ and random correlation matrices, for datasets generated with a specific number of layers ∈{2,…,8}. For each of the number of layers (holding the true values of dyadic and generalized reciprocity matrices fixed), we also plot Frobenius norm estimates from 10 network datasets with differing sample sizes (i.e. number of nodes) ∈{10,…,145}. For each dataset, we fit the multiplex model and calculate the Frobenius norm between the true and estimated dyadic and generalized reciprocity matrices. We visualize these distributions with colour shifting from orange to blue as sample size increases. By comparing the posterior Frobenius norm distributions to the random Frobenius norm distributions, we see that we gain increasingly precise estimates of the true correlation matrices as we increase our sample sizes; our posterior correlation matrices are substantially closer to the true generative correlation matrix than random correlation matrices simulated from the prior, especially as the sample size increases. This indicates that our model is able to accurately recover the key reciprocity parameters used in the true generative model. In the electronic supplementary material, we present similar plots for Poisson and Bernoulli outcome models, which both show the same behaviour as figure 7.

### 6.2. Recovering the effects of predictors

**Figure 8.**
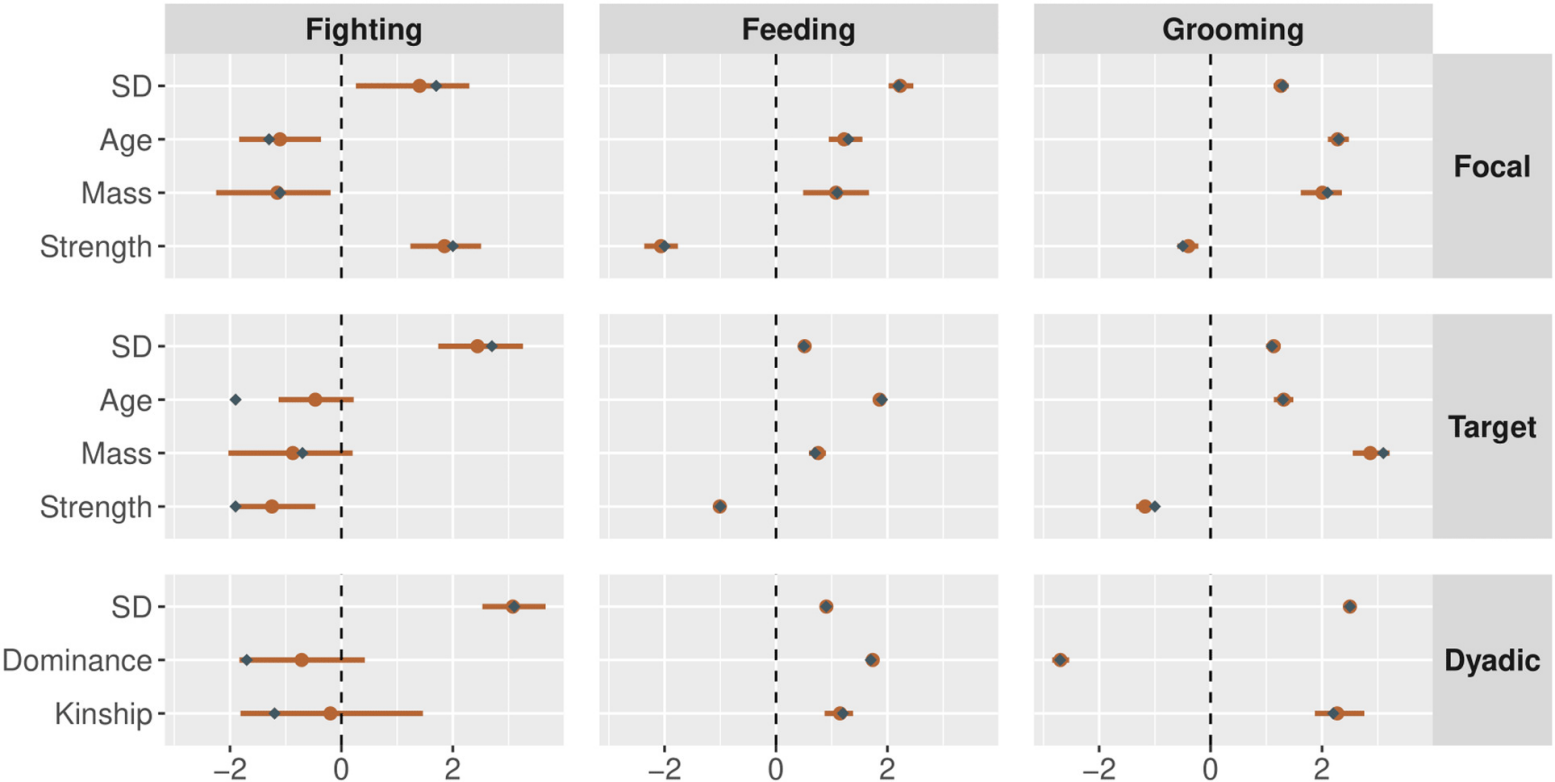
Parameter estimates for the effects of covariates on the structure of network ties in each layer of the simulated data. Orange points represent the posterior medians, and orange bars represent the 90% highest posterior density intervals. Blue diamonds represent the true parameter values used to simulate data. The parameters labelled ‘SD’ are the standard deviations of the random effects. All other estimates are slope coefficients. We observe excellent parameter recovery across all network layers, even the sparse layer (fighting), which was created to be comparably difficult to estimate.

**Figure 9.**
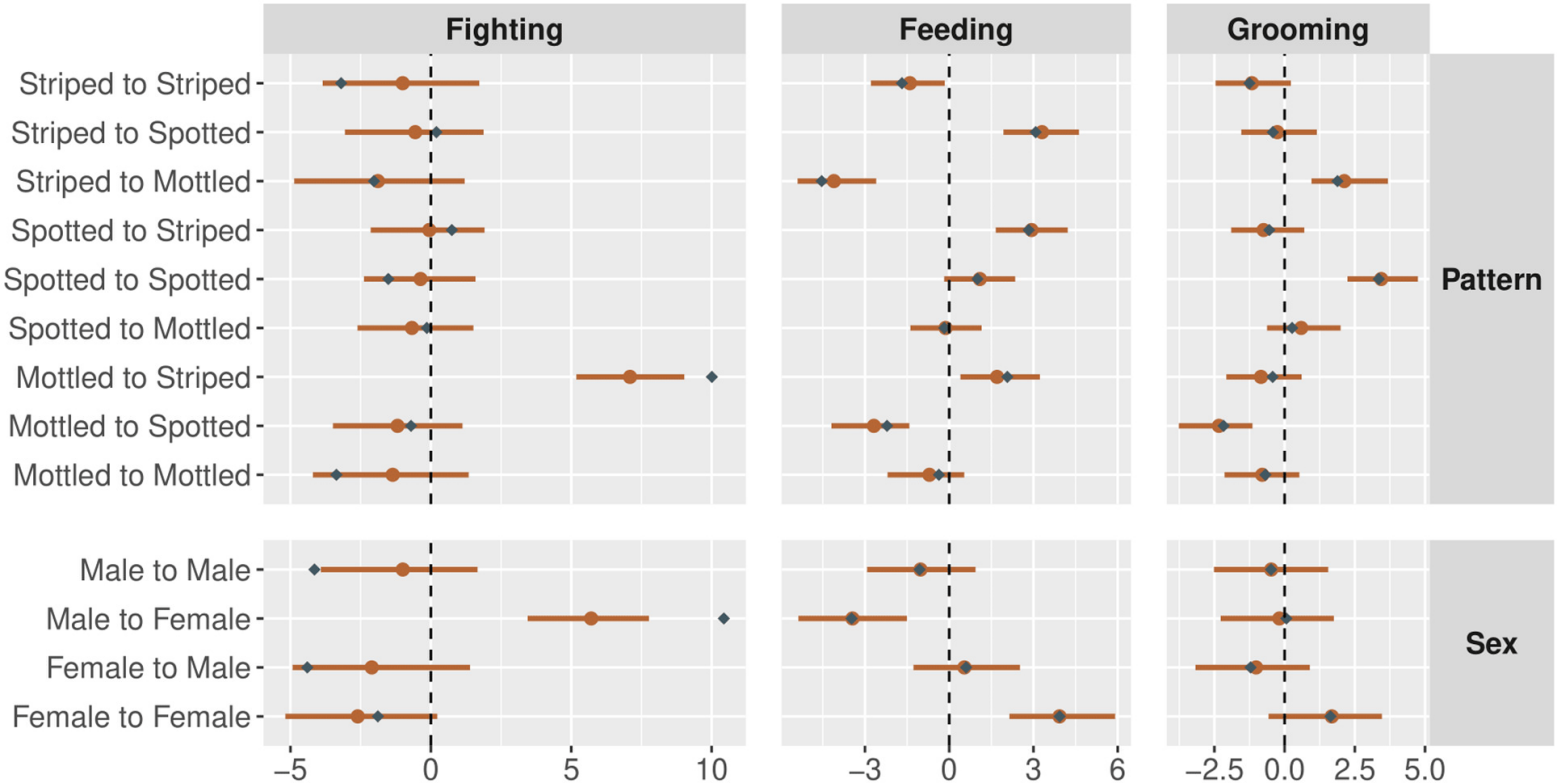
Parameter estimates for the effects of block covariates on the structure of network ties in each layer of the simulated data. Orange points represent the posterior medians, and orange bars represent the 90% highest posterior density intervals. Blue diamonds represent the true parameter values used to simulate data. All estimates are intercept offsets that have been re-centred. We observe excellent parameter recovery across all network layers, but note wider credible regions in the sparse layer (fighting).

**Figure 10.**
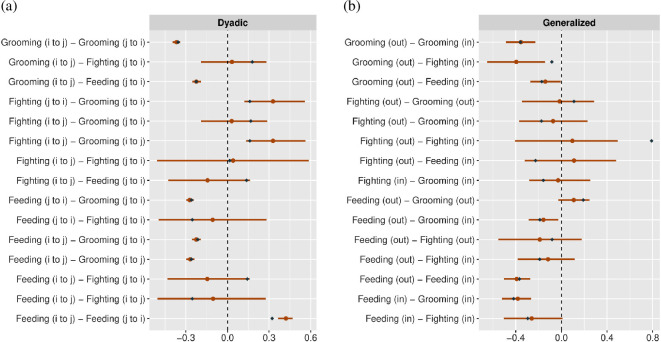
Dyadic (a) and generalized (b) reciprocity estimates, within and between layers, from the simulated dataset. Orange points represent the posterior medians, and orange bars represent the 90% highest posterior density intervals. Blue diamonds represent the true parameter values used to simulate data. We observe excellent parameter recovery, but note wider credible regions for correlations that include the sparse layer (fighting).

Next, we use the simulation engine to create a single three-layer multiplex network, with a full set of focal, target, dyadic and block predictors affecting tie probability within each layer. We simulate data from N=145 nodes and set the parameters such that two layers (feeding and grooming) are fairly dense—and hence are more informative for parameter estimation—and one layer (fighting) is sparse—and hence less informative for parameter estimation. We test the model’s ability to accurately recover the effects of predictor variables by plotting the true generative values and the inferred posterior distributions jointly. Figure 8 plots the focal, target and dyadic predictors in each layer. Figure 9 plots the block predictors in each layer. Figure 10 plots the generalized and dyadic reciprocity effects within and between layers.

Across figures 8–10, we find that we recover not just the correct direction of effects, but precisely correct quantitative estimates for almost all model parameters. These estimates suggest that the model is well specified and capable of supporting robust inference.

## 7. Discussion

Network-based studies of friendship, cooperation, social support, partner choice, norm diffusion, disease diffusion, social learning, economic outcomes and even spiteful behaviours like exploitation and costly reduction have been the focus of much recent work in the social sciences (e.g. [30,38,56,57,63–77]). Similar studies of network structure and its causes and consequences have been conducted in the biological sciences, with case studies spanning study systems from honey bees to vampire bats and killer whales (e.g. [60,78–91]). Increasingly, researchers are advocating moving beyond standard, single-layer, or even layer by layer, modelling approaches, and towards the development and implementation of multiplex or multi-layer network models (e.g. [20,26–28,92]).

Our work here has sought to advance the tools available to end-users so that they can begin to integrate multiplex network approaches into their science. The multiplex tools that we have added to STRAND should be of relevance to both theoreticians and empiricists alike. The STRAND multiplex simulation engine facilitates theoretical studies of the structure of multiplex networks and their coupling or co-evolution with other variables of interest (e.g. [93–100]). The STRAND multiplex data analysis pipeline facilitates empirical studies of multiplex network structure, allowing end-users to deploy powerful Bayesian models to their own data, using only simple base-R modelling syntax.

### 7.1. Conclusions

The STRAND package was initially developed to run Bayesian latent network models on binary, double-sampled outcomes [40] and was later extended to include single-layer networks and Gaussian, Binomial and Poisson outcomes through extensions of the SRM [50]. Here, we have further extended the functionality of STRAND to support the modelling of multiplex networks. We provide both the mathematical formalism underlying the multiplex models and user-friendly tutorials demonstrating how to apply the models on openly available example datasets from both the social and biological sciences. We have also provided detailed unit tests, in order to show that the software performs correctly and allows users to recover the parameters used to simulate artificial datasets. We hope that this extension to the STRAND software package will be of use to empirically minded researchers with limited programming experience. As noted in Ross *et al.* [50], end-users can find a complete index of—and full documentation for—all functions included in STRAND by visiting: https://github.com/ctross/STRAND or by calling ?STRAND from R.

### 7.2. Future directions

STRAND is a software package that is still in active development, with new features being added on a regular basis. We are currently developing an extension of the multiplex model to longitudinal networks [101], integrating automatic Bayesian imputation of missing data, and developing additional custom models—e.g. mixed-membership block models [102], models to facilitate using node-level random effects to predict downstream outcomes, models of network-based diffusion [103], models of experience weighted attraction [82] and models for dimension reduction from multiplex networks to a single-layer latent network. STRAND is community-driven and community-supported. If you have a feature request, please open an issue on our GitHub: https://github.com/ctross/STRAND/issues. Additionally, users can contribute new functionality to STRAND and help in other aspects of development; just open a pull request: https://github.com/ctross/STRAND/pulls and your code will be reviewed and possibly integrated into STRAND, pending quality checks.

## Data Availability

All data used in these examples, as well as all of the code needed to reproduce our results and figures, are included in the GitHub repository where the R package is hosted: https: //github.com/ctross/STRAND. Supplementary material is available online [104].

## References

[1] Rhodes DR, Tomlins SA, Varambally S, Mahavisno V, Barrette T, Kalyana-Sundaram S, Ghosh D, Pandey A, Chinnaiyan AM. 2005 Probabilistic model of the human protein-protein interaction network. Nat. Biotechnol. **23**, 951–959. (10.1038/nbt1103) 16082366

[2] Zengler K, Zaramela LS. 2018 The social network of microorganisms—how auxotrophies shape complex communities. Nat. Rev. Microbiol. **16**, 383–390. (10.1038/s41579-018-0004-5) 29599459 PMC6059367

[3] Snyder-Mackler N *et al*. 2020 Social determinants of health and survival in humans and other animals. Science **368**, x9553. (10.1126/science.aax9553) PMC7398600 32439765

[4] Croft DP, James R, Krause J. 2008 Exploring animal social networks. Princeton, NJ: Princeton University Press.

[5] Kajokaite K, Whalen A, Koster J, Perry S. 2022 Social integration predicts survival in female white-faced capuchin monkeys. Behav. Ecol. **33**, 807–815. (10.1093/beheco/arac043) 35812363 PMC9262163

[6] Buldyrev SV, Parshani R, Paul G, Stanley HE, Havlin S. 2010 Catastrophic cascade of failures in interdependent networks. Nature **464**, 1025–1028. (10.1038/nature08932) 20393559

[7] Jackson MO. 2011 An overview of social networks and economic applications. In Handbook of social economics (eds J Benhabib, A Bisin, MO Jackson), pp. 511–585. San Diego, CA: Elsevier. (10.1016/b978-0-444-53187-2.00012-7)

[8] Borgatti SP, Mehra A, Brass DJ, Labianca G. 2009 Network analysis in the social sciences. Science **323**, 892–895. (10.1126/science.1165821) 19213908

[9] Snijders TAB, van de Bunt GG, Steglich CEG. 2010 Introduction to stochastic actor-based models for network dynamics. Soc. Networks **32**, 44–60. (10.1016/j.socnet.2009.02.004)

[10] Krivitsky PN. 2012 Exponential-family random graph models for valued networks. Electron. J. Stat. **6**. (10.1214/12-ejs696) PMC3964598 24678374

[11] Borgatti SP, Everett MG, Johnson JC, Agneessens F. 2022 Analyzing social networks using R. London, UK: Sage Publications.

[12] Robins G, Snijders T, Wang P, Handcock M, Pattison P. 2007 Recent developments in exponential random graph (p*) models for social networks. Soc. Networks **29**, 192–215. (10.1016/j.socnet.2006.08.003)

[13] Kenny DA, La Voie L. 1984 The social relations model. In Advances in experimental social psychology (ed. L Berkowitz), pp. 141–182. Oxford, UK: Academic Press. (10.1016/S0065-2601(08)60144-6)

[14] Back MD, Kenny DA. 2010 The social relations model: how to understand dyadic processes. Soc. Personal. Psychol. Compass **4**, 855–870. (10.1111/j.1751-9004.2010.00303.x)

[15] Koster J, Leckie G, Aven B. 2020 Statistical methods and software for the multilevel social relations model. Field Methods **32**, 339–345. (10.1177/1525822x19889011)

[16] Holland PW, Laskey KB, Leinhardt S. 1983 Stochastic blockmodels: first steps. Soc. Networks **5**, 109–137. (10.1016/0378-8733(83)90021-7)

[17] Newman MEJ. 2006 Modularity and community structure in networks. Proc. Natl Acad. Sci. USA **103**, 8577–8582. (10.1073/pnas.0601602103) 16723398 PMC1482622

[18] Fisher DN, Pinter-Wollman N. 2021 Using multilayer network analysis to explore the temporal dynamics of collective behavior. Curr. Zool. **67**, 71–80. (10.1093/cz/zoaa050) 33654492 PMC7901757

[19] Snijders TAB. 2011 Statistical models for social networks. Annu. Rev. Sociol. **37**, 131–153. (10.1146/annurev.soc.012809.102709)

[20] Atkisson C, Górski PJ, Jackson MO, Hołyst JA, D’Souza RM. 2020 Why understanding multiplex social network structuring processes will help us better understand the evolution of human behavior. Evol. Anthropol. **29**, 102–107. (10.1002/evan.21850) 32544306

[21] Farine DR, Whitehead H. 2015 Constructing, conducting and interpreting animal social network analysis. J. Anim. Ecol. **84**, 1144–1163. (10.1111/1365-2656.12418) 26172345 PMC4973823

[22] Sosa S, Sueur C, Puga‐Gonzalez I. 2021 Network measures in animal social network analysis: their strengths, limits, interpretations and uses. Methods Ecol. Evol. **12**, 10–21. (10.1111/2041-210x.13366)

[23] Kivela M, Arenas A, Barthelemy M, Gleeson JP, Moreno Y, Porter MA. 2014 Multilayer networks. J. Complex Netw. **2**, 203–271. (10.1093/comnet/cnu016)

[24] Boccaletti S, Bianconi G, Criado R, del Genio CI, Gómez-Gardeñes J, Romance M, Sendiña-Nadal I, Wang Z, Zanin M. 2014 The structure and dynamics of multilayer networks. Phys. Rep. **544**, 1–122. (10.1016/j.physrep.2014.07.001) 32834429 PMC7332224

[25] Beisner B, Braun N, Pósfai M, Vandeleest J, D’Souza R, McCowan B. 2020 A multiplex centrality metric for complex social networks: sex, social status, and family structure predict multiplex centrality in rhesus macaques. PeerJ **8**, e8712. (10.7717/peerj.8712) 32211232 PMC7081788

[26] Silk MJ *et al*. 2018 Quantifying direct and indirect contacts for the potential transmission of infection between species using a multilayer contact network. Behaviour **155**, 731–757. (10.1163/1568539x-00003493)

[27] Silk MJ, Finn KR, Porter MA, Pinter-Wollman N. 2018 Can multilayer networks advance animal behavior research? Trends Ecol. Evol. **33**, 376–378. (10.1016/j.tree.2018.03.008) 29685580 PMC5962412

[28] Dickison ME, Magnani M, Rossi L. 2016 Multilayer social networks. Cambridge, UK: Cambridge University Press.

[29] Artime O, Benigni B, Bertagnolli G, d’Andrea V, Gallotti R, Ghavasieh A, Raimondo S, Domenico M. 2022 Multilayer network science: from cells to societies. Cambridge, UK: Cambridge University Press.

[30] Redhead D, Gervais M, Kajokaite K, Koster J, Hurtado Manyoma A, Hurtado Manyoma D, McElreath R, Ross CT. 2024 Evidence of direct and indirect reciprocity in network-structured economic games. Commun. Psychol. **2**, 44. (10.1038/s44271-024-00098-1) 39242753 PMC11332088

[31] Hong A, Niezink NMD. 2024 The multiplex p2 model: mixed-effects modeling for multiplex social networks. Bayesian Anal. **1**, 1–31. (10.1214/25-BA1527)

[32] De Domenico M. 2022 Multilayer networks: analysis and visualization. Introduction to muxViz with R. Cham, Switzerland: Springer. (10.1007/978-3-030-75718-2)

[33] Sosa S, Brooke McElreath M, Redhead D, Ross CT. 2025 Robust Bayesian analysis of animal networks subject to biases in sampling intensity and censoring. Methods Ecol. Evol. **16**, 1273–1294. (10.1111/2041-210X.70017)

[34] Weiss MN, Franks DW, Brent LJN, Ellis S, Silk MJ, Croft DP. 2021 Common datastream permutations of animal social network data are not appropriate for hypothesis testing using regression models. Methods Ecol. Evol. **12**, 255–265. (10.1111/2041-210x.13508) 35464674 PMC9033095

[35] Puga‐Gonzalez I, Sueur C, Sosa S. 2021 Null models for animal social network analysis and data collected via focal sampling: pre‐network or node network permutation? Methods Ecol. Evol. **12**, 22–32. (10.1111/2041-210x.13400)

[36] Hart JDA, Weiss MN, Brent LJN, Franks DW. 2022 Common permutation methods in animal social network analysis do not control for non-independence. Behav. Ecol. Sociobiol. **76**, 151. (10.1007/s00265-022-03254-x) 36325506 PMC9617964

[37] Farine DR, Carter GG. 2022 Permutation tests for hypothesis testing with animal social network data: problems and potential solutions. Methods Ecol. Evol. **13**, 144–156. (10.1111/2041-210x.13741) 35873757 PMC9297917

[38] Koster JM, Leckie G. 2014 Food sharing networks in lowland Nicaragua: an application of the social relations model to count data. Soc. Networks **38**, 100–110. (10.1016/j.socnet.2014.02.002)

[39] McElreath R. 2020 Statistical rethinking: a Bayesian course with examples in R and Stan. Boca Raton, FL: CRC Press.

[40] Redhead D, McElreath R, Ross CT. 2024 Reliable network inference from unreliable data: a tutorial on latent network modeling using STRAND. Psychol. Methods **29**, 1100–1122. (10.1037/met0000519) 36877490

[41] Hahn ED, Soyer R. 2005 Probit and logit models: differences in the multivariate realm.

[42] Newman MEJ. 2003 Mixing patterns in networks. Phys. Rev. E **67**, 026126. (10.1103/physreve.67.026126) 12636767

[43] Papaspiliopoulos O, Roberts GO, Sköld M. 2007 A general framework for the parametrization of hierarchical models. Stat. Sci. 59–7322. **22**. (10.1214/088342307000000014)

[44] Pinkney S. 2024 A short note on a flexible Cholesky parameterization of correlation matrices. arxiv 2405.07286. https://arxiv.org/abs/2405.07286

[45] Pinkney S, Ross C. 2025 Generating positive-definite correlation matrices with additional symmetries. OSF. (10.31235/osf.io/ftvxe_v1)

[46] Sosa S, McElreath MB, Redhead D, Ross CT. 2025 Robust Bayesian analysis of animal networks subject to biases in sampling intensity and censoring. Methods Ecol. Evol. **16**, 1273–1294. (10.1111/2041-210X.70017)

[47] Hoff PD. 2015 Dyadic data analysis with amen. arxiv 1506.08237. https://arxiv.org/abs/1506.08237

[48] Koster J, Leckie G, Aven B, Charlton C. 2020 Statistical methods and software for the multilevel social relations model. Field Methods **32**, 339–345. (10.1177/1525822X19889011)

[49] Snijders TAB, Kenny DA. 1999 The social relations model for family data: a multilevel approach. Pers. Relatsh. **6**, 471–486. (10.1111/j.1475-6811.1999.tb00204.x)

[50] Ross CT, McElreath R, Redhead D. 2024 Modelling animal network data in R using STRAND. J. Anim. Ecol. **93**, 254–266. (10.1111/1365-2656.14021) 37936514

[51] Ready E, Power EA. 2021 Measuring reciprocity: double sampling, concordance, and network construction. Netw. Sci. **9**, 387–402. (10.1017/nws.2021.18)

[52] Gelman A, Carlin JB, Stern HS, Rubin DB. 1995 Bayesian data analysis. Boca Raton, FL: Chapman and Hall/CRC.

[53] Stan Development Team. 2021 CmdStanR: a lightweight interface to Stan for R users. See https://github.com/stan-dev/cmdstanr.

[54] Stan Development Team. 2021 Stan modeling language users guide and reference manual. See https://mc-stan.org.

[55] Gervais MM. 2017 RICH economic games for networked relationships and communities: development and preliminary validation in Yasawa, Fiji. Field Methods **29**, 113–129. (10.1177/1525822x16643709)

[56] Pisor AC, Gervais MM, Purzycki BG, Ross CT. 2020 Preferences and constraints: the value of economic games for studying human behaviour. R. Soc. Open Sci. **7**, 192090. (10.1098/rsos.192090) 32742683 PMC7353969

[57] Ross CT, Pisor AC. 2024 Perceived inequality and variability in the expression of parochial altruism. Cambridge, UK: Cambridge University Press. (10.1017/ehs.2024.43) PMC11810521 39935444

[58] Koster J, Leckie G, Miller A, Hames R. 2015 Multilevel modeling analysis of dyadic network data with an application to Ye’kwana food sharing. Am. J. Phys. Anthropol. **157**, 507–512. (10.1002/ajpa.22721) 25773376

[59] Bhui R, Chudek M, Henrich J. 2019 How exploitation launched human cooperation. Behav. Ecol. Sociobiol **73**, 1–14. (10.1007/s00265-019-2667-y)

[60] Gelardi V, Fagot J, Barrat A, Claidière N. 2019 Detecting social (in)stability in primates from their temporal co-presence network. Anim. Behav. **157**, 239–254. (10.1016/j.anbehav.2019.09.011)

[61] Gelardi V, Godard J, Paleressompoulle D, Claidiere N, Barrat A. 2020 Measuring social networks in primates: wearable sensors versus direct observations. Proc. R. Soc. A **476**, 20190737. (10.1098/rspa.2019.0737) 32398933 PMC7209153

[62] Cui X, Li C, Zhao J, Zeng L, Zhang D, Pan J. 2016 Covariance structure regularization via Frobenius-norm discrepancy. Linear Algebr. Its Appl. **510**, 124–145. (10.1016/j.laa.2016.08.013)

[63] Nolin DA. 2010 Food-sharing networks in Lamalera, Indonesia. Hum. Nat. **21**, 243–268. (10.1007/s12110-010-9091-3) 21218145 PMC3014574

[64] Apicella CL, Marlowe FW, Fowler JH, Christakis NA. 2012 Social networks and cooperation in hunter-gatherers. Nature **481**, 497–501. (10.1038/nature10736) 22281599 PMC3340565

[65] Kasper C, Mulder MB. 2015 Who helps and why? Curr. Anthropol. **56**, 701–732. (10.1086/683024)

[66] Crittenden AN, Zes DA. 2015 Food sharing among Hadza hunter-gatherer children. PLoS One **10**, e0131996. (10.1371/journal.pone.0131996) 26151637 PMC4494808

[67] Colleran H, Mace R. 2015 Social network- and community-level influences on contraceptive use: evidence from rural Poland. Proc. R. Soc. B **282**, 20150398. (10.1098/rspb.2015.0398) PMC4424654 25904669

[68] Power EA. 2017 Social support networks and religiosity in rural South India. Nat. Hum. Behav. **1**. (10.1038/s41562-017-0057)

[69] Ready E, Power EA. 2018 Why wage earners hunt: food sharing, social structure, and influence in an Arctic mixed economy. Curr. Anthropol. **59**, 74–97. (10.1086/696018)

[70] Ziker JP, Fulk KS. 2018 Indigenous Siberian food sharing networks: social innovation in a transforming economy. In Collaborative innovation networks (eds F Grippa, J Leitão, J Gluesing, K Riopelle, P Gloor), pp. 117–127. Cham, Switzerland: Springer. (10.1007/978-3-319-74295-3_10)

[71] Campbell EM *et al*. 2017 Detailed transmission network analysis of a large opiate-driven outbreak of HIV infection in the United States. J. Infect. Dis. **216**, 1053–1062. (10.1093/infdis/jix307) 29029156 PMC5853229

[72] Starkweather KE, Reynolds AZ, Zohora F, Alam N. 2023 Shodagor women cooperate across domains of work and childcare to solve an adaptive problem. Phil. Trans. R. Soc. B **378**, 20210433. (10.1098/rstb.2021.0433) 36440563 PMC9703234

[73] Valle Nunes A, Guariento RD, Santos BA, Fischer E. 2019 Wild meat sharing among non-indigenous people in the southwestern Amazon. Behav. Ecol. Sociobiol. **73**, 26. (10.1007/s00265-018-2628-x)

[74] Koster J *et al*. 2019 Kinship ties across the lifespan in human communities. Phil. Trans. R. Soc. B **374**, 20180069. (10.1098/rstb.2018.0069) 31303163 PMC6664140

[75] Minocher R, Ross CT. 2022 Spousal age-gaps, partner preferences, and consequences for well-being in four Colombian communities. Evol. Hum. Behav. **43**, 394–407. (10.1016/j.evolhumbehav.2022.06.004)

[76] Redhead D, Ragione AD, Ross CT. 2023 Friendship and partner choice in rural Colombia. Evol. Hum. Behav. **44**, 2022. (10.1016/j.evolhumbehav.2022.08.004)

[77] Jackson MO, Rogers BW, Zenou Y. 2017 The economic consequences of social-network structure. J. Econ. Lit. **55**, 49–95. (10.1257/jel.20150694)

[78] Gómez JM, Nunn CL, Verdú M. 2013 Centrality in primate–parasite networks reveals the potential for the transmission of emerging infectious diseases to humans. Proc. Natl Acad. Sci. USA **110**, 7738–7741. (10.1073/pnas.1220716110) 23610389 PMC3651426

[79] Wild B, Dormagen DM, Zachariae A, Smith ML, Traynor KS, Brockmann D, Couzin ID, Landgraf T. 2021 Social networks predict the life and death of honey bees. Nat. Commun. **12**, 1110. (10.1038/s41467-021-21212-5) 33597518 PMC7889932

[80] Lusseau D. 2003 The emergent properties of a dolphin social network. Proc. R. Soc. B **270**, S186–S188. (10.1098/rsbl.2003.0057) PMC1809954 14667378

[81] Wiszniewski J, Lusseau D, Möller LM. 2010 Female bisexual kinship ties maintain social cohesion in a dolphin network. Anim. Behav. **80**, 895–904. (10.1016/j.anbehav.2010.08.013)

[82] Barrett BJ, McElreath RL, Perry SE. 2017 Pay-off-biased social learning underlies the diffusion of novel extractive foraging traditions in a wild primate. Proc. R. Soc. B **284**, 20170358. (10.1098/rspb.2017.0358) PMC5474070 28592681

[83] Ripperger SP *et al*. 2019 Vampire bats that cooperate in the lab maintain their social networks in the wild. Curr. Biol. **29**, 4139–4144.(10.1016/j.cub.2019.10.024) 31679938

[84] Aplin LM, Farine DR, Morand-Ferron J, Cockburn A, Thornton A, Sheldon BC. 2015 Experimentally induced innovations lead to persistent culture via conformity in wild birds. Nature **518**, 538–541. (10.1038/nature13998) 25470065 PMC4344839

[85] Guimarães PR, de Menezes MA, Baird RW, Lusseau D, Guimarães P, dos Reis SF. 2007 Vulnerability of a killer whale social network to disease outbreaks. Phys. Rev. E **76**, 042901. (10.1103/physreve.76.042901) 17995045

[86] Madden JR, Drewe JA, Pearce GP, Clutton-Brock TH. 2009 The social network structure of a wild meerkat population: 2. Intragroup interactions. Behav. Ecol. Sociobiol. **64**, 81–95. (10.1007/s00265-009-0820-8)

[87] Drewe JA. 2010 Who infects whom? Social networks and tuberculosis transmission in wild meerkats. Proc. R. Soc. B **277**, 633–642. (10.1098/rspb.2009.1775) PMC2842696 19889705

[88] Silk JB, Beehner JC, Bergman TJ, Crockford C, Engh AL, Moscovice LR, Wittig RM, Seyfarth RM, Cheney DL. 2010 Strong and consistent social bonds enhance the longevity of female baboons. Curr. Biol. **20**, 1359–1361. (10.1016/j.cub.2010.05.067) 20598541

[89] DeTroy SE, Ross CT, Cronin KA, van Leeuwen EJC, Haun DBM. 2021 Cofeeding tolerance in chimpanzees depends on group composition: a longitudinal study across four communities. iScience **24**, 102453. (10.1016/j.isci.2021.102453) 33997714 PMC8105662

[90] Ilany A, Holekamp KE, Akçay E. 2021 Rank-dependent social inheritance determines social network structure in spotted hyenas. Science **373**, 348–352. (10.1126/science.abc1966) 34437155

[91] Carter GG, Wilkinson GS. 2013 Food sharing in vampire bats: reciprocal help predicts donations more than relatedness or harassment. Proc. R. Soc. B **280**, 20122573. (10.1098/rspb.2012.2573) PMC3574350 23282995

[92] Hasenjager MJ, Silk M, Fisher DN. 2021 Multilayer network analysis: new opportunities and challenges for studying animal social systems. Curr. Zool. **67**, 45–48. (10.1093/cz/zoab006) 33654489 PMC7901768

[93] Smolla M, Akçay E. 2019 Cultural selection shapes network structure. Sci. Adv. **5**, eaaw0609. (10.1126/sciadv.aaw0609) 31453324 PMC6693906

[94] Yeh DJ, Fogarty L, Kandler A. 2019 Cultural linkage: the influence of package transmission on cultural dynamics. Proc. R. Soc. B **286**, 20191951. (10.1098/rspb.2019.1951) PMC6939269 31795868

[95] Snijders T, Steglich C, Schweinberger M. 2017 Modeling the coevolution of networks and behavior. In Longitudinal models in the behavioral and related sciences (eds K van Montfort, J Oud, A Satorra), pp. 41–71. New York, NY: Routledge. (10.4324/9781315091655-3)

[96] Jackson MO, Yariv L. 2007 Diffusion of behavior and equilibrium properties in network games. Am. Econ. Rev. **97**, 92–98. (10.1257/aer.97.2.92)

[97] Lazer D, Rubineau B, Chetkovich C, Katz N, Neblo M. 2010 The coevolution of networks and political attitudes. Polit. Commun. **27**, 248–274. (10.1080/10584609.2010.500187)

[98] Arifovic J, Eaton BC, Walker G. 2015 The coevolution of beliefs and networks. J. Econ. Behav. Organ. **120**, 46–63. (10.1016/j.jebo.2015.08.011)

[99] Wu B, Zhou D, Fu F, Luo Q, Wang L, Traulsen A. 2010 Evolution of cooperation on stochastic dynamical networks. PLoS One **5**, e11187. (10.1371/journal.pone.0011187) 20614025 PMC2894855

[100] Bowles S, Gintis H. 2004 Persistent parochialism: trust and exclusion in ethnic networks. J. Econ. Behav. Organ. **55**, 1–23. (10.1016/j.jebo.2003.06.005)

[101] Ross C, Pinkney S, McElreath R. 2025 Bayesian longitudinal social network models: an implementation in R using STRAND. Center for Open Science. (10.31235/osf.io/ghwts_v1)

[102] Airoldi EM, Blei DM, Erosheva EA, Fienberg SE. 2014 Introduction to mixed membership models and methods. In Handbook of mixed membership models and their applications, pp. 3–14. Boca Raton, FL: Chapman & Hall.

[103] Franz M, Nunn CL. 2009 Network-based diffusion analysis: a new method for detecting social learning. Proc. R. Soc. B **276**, 1829–1836. (10.1098/rspb.2008.1824) PMC2674490 19324789

[104] Ross CT, Kajokait K, Pinkney S, Sosa S. 2025 Supplementary material from: Bayesian multiplex network models in R using STRAND: Methods for biologists and social scientists. Figshare. (10.6084/m9.figshare.c.8054238) PMC12539975 41127800

